# Comprehensive genomic profiling of triple-negative breast cancer metastases identifies role of PKD1 in immunotherapy resistance

**DOI:** 10.1172/JCI188989

**Published:** 2026-03-02

**Authors:** Xiu-Zhi Zhu, Yi-Fan Zhou, Xiao-Han Ying, Yun-Yi Wang, Xiao-Hong Ding, Kun-Yu Zhang, Zhi-Ming Shao, Xi Jin, Yi-Zhou Jiang, Zhong-Hua Wang

**Affiliations:** 1 Department of Breast Surgery, Fudan University Shanghai Cancer Center, Shanghai, China.; 2 Key Laboratory of Breast Cancer in Shanghai, Department of Oncology, Shanghai Medical College, Fudan University, Shanghai, China.

**Keywords:** Genetics, Oncology, Breast cancer, Immunotherapy

## Abstract

The multi-omics data represented by genomic data from patients with metastatic triple-negative breast cancer (TNBC) is crucial for precision treatment, yet data on genomic alterations in metastatic cohorts and Chinese populations remains limited. We performed targeted sequencing of 296 metastatic TNBC samples from 296 patients treated at Fudan University Shanghai Cancer Center (October 2018 to November 2020) using a 484-gene panel, identifying 796 metastatic events across 18 organ sites. We characterized the genomic landscape of TNBC metastases and identified marked enrichment of polycystin-1 (PKD1) mutations in metastatic lesions — a finding validated in an independent paired primary metastasis cohort (*n* = 105). Notably, *PKD1* mutations were associated with resistance to anti–PD-1 therapy, as validated across 3 clinical trials (NCT03805399, NCT04129996, and NCT04395989). Multi-omics analyses, combined with functional in vitro and in vivo mechanistic studies, revealed that PKD1 modulated the “desert” tumor immune microenvironment via C-C motif chemokine ligand 2 (CCL2), and targeting CCL2 could reverse immunotherapy resistance. This comprehensive genomic characterization of metastases enhances our understanding of tumor evolution, identifies PKD1 as a previously uncharacterized regulator of immune evasion to our knowledge, and suggests a potential therapeutic strategy to overcome immunotherapy resistance.

## Abstract 2

Large-scale DNA sequencing reveals genomic characterization of triple-negative breast cancer metastases and uncovers PKD1’s role in immunotherapy resistance.

## Introduction

Triple-negative breast cancer (TNBC) accounts for approximately 15% of all breast cancer cases and is characterized by the absence of estrogen receptor (ER), progesterone receptor (PR), and human epidermal growth factor receptor 2 (HER2) expression (1). Compared with other breast cancer subtypes, TNBC is associated with an unfavorable prognosis, marked by an increased risk of early recurrence and distant metastasis (2). The 5-year disease-free survival rate for early-stage TNBC remains approximately 80% (3), and metastatic TNBC (mTNBC) confers a particularly poor outcome, with a median overall survival of only 17–27 months after metastasis detection (4–7). Furthermore, TNBC exhibits high molecular and clinical heterogeneity and can be classified into several distinct subtypes — such as basal-like immune-suppressed, luminal androgen receptor, and immunomodulatory — each demonstrating unique biological behavior and therapeutic responses (4, 8, 9). Considering the unfavorable prognosis and high heterogeneity, prioritizing mTNBC research to improve patient outcomes is crucial.

The treatment landscape for TNBC is evolving. The approval of immune checkpoint inhibitors (10), poly (ADP-ribose) polymerase (PARP) inhibitors for patients with germline breast cancer susceptibility gene (BRCA)1 and BRCA2 mutations (11), and antibody-drug conjugates like sacituzumab govitecan has provided new options for patients with TNBC (12). However, effective strategies remain limited for the majority of patients with mTNBC due to the lack of actionable targets and primary or acquired resistance. Consequently, chemotherapy remains a therapeutic backbone. This underscores the critical need to prioritize research aimed at identifying novel predictive biomarkers and therapeutic targets to improve treatment efficacy.

Genomic precision medicine has yielded promising outcomes for patients with TNBC, underscoring the importance of genomic research in TNBC. The PARP inhibitor olaparib, which targets *BRCA1* or *BRCA2* mutations, significantly improved disease-free survival in patients in the OlympiA phase III trial (13). In addition, the AKT inhibitor capivasertib, when combined with first-line paclitaxel therapy for TNBC in the PAKT phase II trial, resulted in significantly longer progression-free survival and overall survival in patients with *PIK3CA/AKT1/PTEN*-altered tumors (14), which directly inspired the subsequent phase III investigation (CAPItello-290 trial). Each of these studies underscores the importance of investigating genomic profiles to advance the comprehension and precision treatment of TNBC.

While primary breast cancer has been intensely studied (15), the genomic and phenotypic alterations that arise during tumor progression and metastatic colonization remain incompletely characterized, especially for mTNBC. Longitudinal analyses have revealed that receptor expression (such as ER and PR) can shift between primary breast cancers and their matched metastases (16, 17). Recent multiregion sequencing reveals that new driver mutations frequently emerge in distant metastases but not in primary breast cancers (18, 19). Similar patterns of metastasis-restricted mutations have been documented in colorectal cancer (20). These findings underscore the importance of genomic analysis in metastatic disease. Pioneering studies in characterizing the genomic landscape of cancer metastasis have provided crucial insights into tumor evolution and potential therapeutic targets (21–26). Overall, the genomic information obtained from the primary tumor is insufficient to fully guide the treatment of patients with metastatic cancer. Considering the racial differences of participants (27, 28) and limited sample sizes examined (19, 29–31) in previous studies, it is necessary to conduct a large-scale genomic study of mTNBC in a Chinese population.

To conduct a comprehensive and in-depth exploration of the genomic characteristics of mTNBC, we established a large-scale cohort comprising 296 patients with TNBC metastases. Pathologically confirmed metastatic samples from these patients were subjected to targeted next-generation sequencing (NGS) using a panel of 484 breast cancer–associated genes. Our study highlights the clinical value of the genomic landscape of TNBC metastases, providing directions for future precision treatment strategies for mTNBC.

## Results

### Study design and clinical cohort.

**Figure 1:**
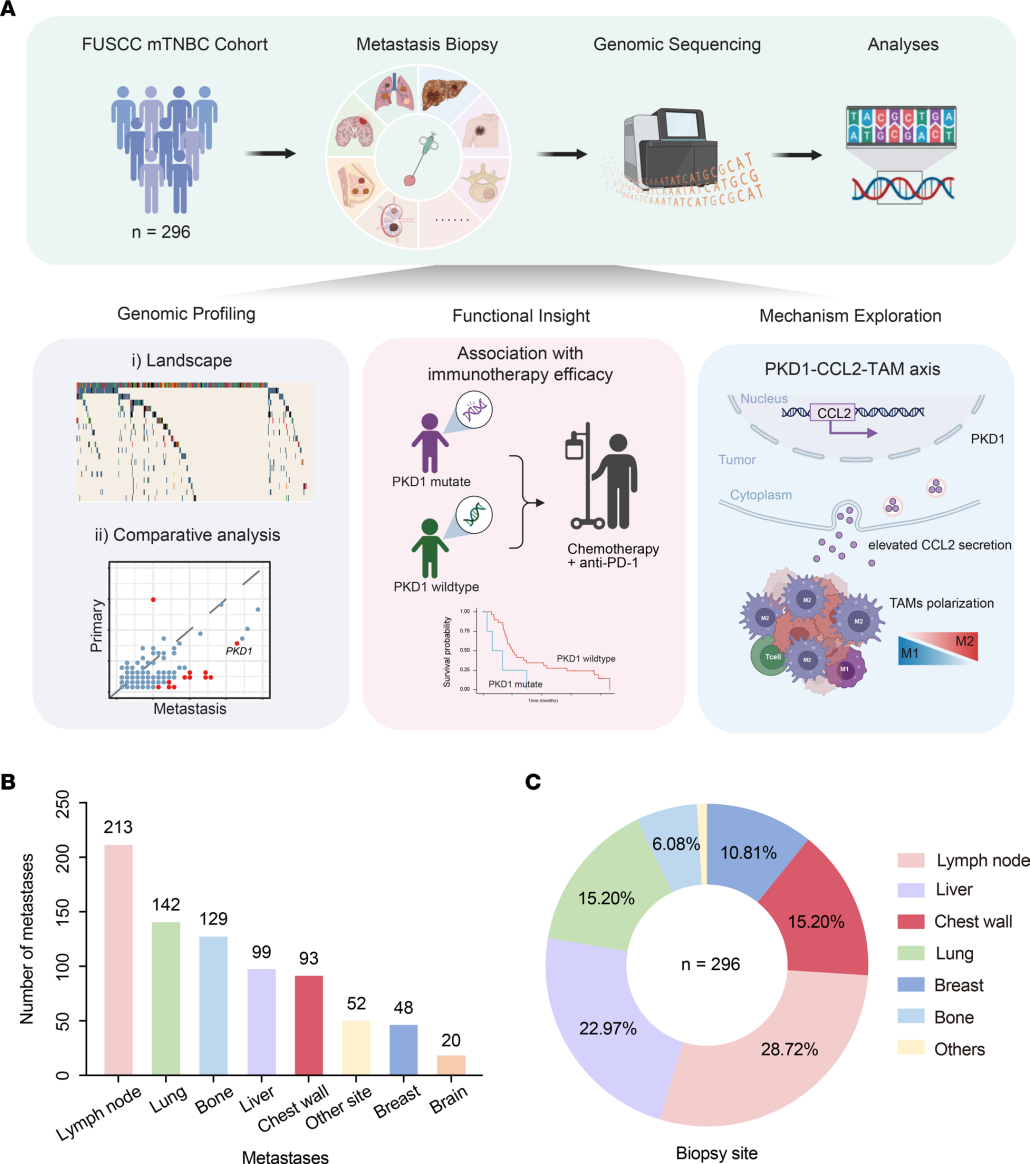
Schematic of the study and sample distribution. (**A**) Schematic overview of the study design. NGS was conducted to analyze the genomic profiles of TNBC metastases. Genomic landscape differences of TNBC metastases were assessed across the following categories: TNBC metastases from Western and Chinese populations; primary tumors and metastatic tumors; and metastases occurring in different organs. Personalized treatment regimens were administered to these patients on the basis of the NGS results. Efficacy analysis was performed to identify potential biomarkers associated with specific treatment responses. Mechanistic research was conducted to reveal the underlying mechanisms of specific biomarkers. (**B**) Distribution of 796 metastatic events across 18 organ sites in patients with advanced TNBC. Other sites (11 total) include pleura, adrenal gland, abdominal wall, and additional locations with lower metastatic frequency. (**C**) Distribution of 296 advanced TNBC biopsy sites in our FUSCC cohort. NGS, next-generation sequencing; TNBC, triple-negative breast cancer; mTNBC, metastatic triple-negative breast cancer; FUSCC, Fudan University Shanghai Cancer Center.

To comprehensively describe the genomic characteristics of TNBC metastases, we prospectively collected 296 TNBC metastases and paired peripheral blood samples from patients with mTNBC treated at Fudan University Shanghai Cancer Center (FUSCC) from October 2018 to November 2020 (Supplemental Figure 1, A and B; supplemental material available online with this article; https://doi.org/10.1172/JCI188989DS1B). We performed targeted NGS using the FUSCC breast cancer–associated 484-gene panel (FUSCC-BC gene panel) on all metastases (32), and collected data on somatic mutations and copy number variations (CNVs). We described the genomic differences in TNBC from 3 main aspects: (a) among different metastatic organs, (b) between Chinese and Western populations, and (c) between paired and unpaired primary and metastatic lesions within the FUSCC cohort. Moreover, we analyzed genomic alterations associated with immunotherapy efficacy and explored the underlying mechanisms, highlighting the translational relevance of genomic profiling of metastases (Figure 1A).

A total of 796 metastatic events from 296 patients with mTNBC treated at FUSCC were retrieved and mapped to 18 organ sites. The most common metastatic sites were the lymph nodes (*n* = 213), lungs (*n* = 142), bones (*n* = 129), liver (*n* = 99), and chest wall (*n* = 93) (Figure 1B and Supplemental Figure 1C). The frequencies of organ-specific metastasis in patients with mTNBC were comparable to those reported in previous studies (29). For patients with multiple metastatic events, we selected and sequenced 1 representative metastatic lesion per patient. The distributions and proportions of 296 biopsy sites of metastases are shown in Figure 1C, and the top biopsy sites were the lymph nodes, liver, lungs, and chest wall, which accounted for 28.72%, 22.97%, 15.20%, and 15.20% of biopsies, respectively. Compared with the Western mTNBC clinical cohort investigated at Memorial Sloan Kettering Cancer Center (MSKCC, *n* = 143) (33), our cohort included a significantly higher proportion of heavily pretreated patients, defined as those having received ≥3 lines of systemic therapy for metastatic disease prior to enrollment (27.8% vs. 13.2%; *P* < 0.001, Supplemental Figure 1D). Note that the clinical characteristics of patients with mTNBC in the FUSCC cohort and MSKCC cohort are summarized in Supplemental Figure 1D and Supplemental Table 1.

### Genomic landscape of TNBC metastases.

**Figure 2:**
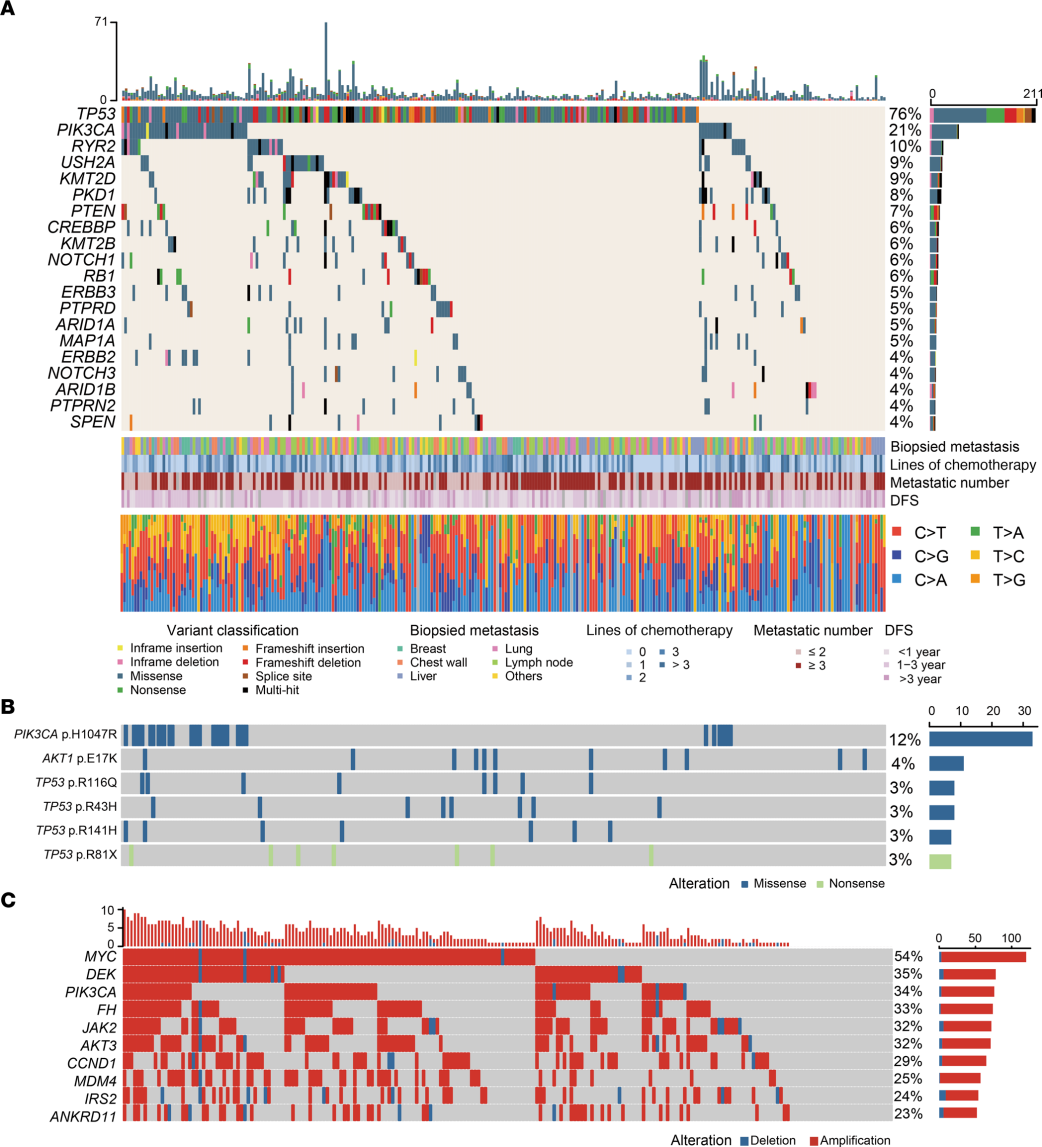
Genomic landscape of triple-negative breast cancer metastases. (**A**) Sequencing data of 296 Chinese mTNBC tissue samples classified by mutation profile and annotated with the variation type and mutation frequency. The mutation counts in each sample and each gene are provided above and on the right side, respectively. (**B**) Hotspot mutations in Chinese mTNBC tissue samples. (**C**) Copy number variations (top 10) of 296 Chinese mTNBC tissue samples in our cohort. DFS, disease-free survival; mTNBC, metastatic triple-negative breast cancer.

We described the genomic landscape of 296 TNBC metastases, including mutant genes, mutation sites, and CNVs (Figure 2). The most prevalent somatic variation of TNBC metastases in our cohort was *TP53* (76%), followed by *PIK3CA* (21%), *RYR2* (10%), *USH2A* (9%), *KMT2D* (9%) and *PKD1* (8%) (Figure 2A). Comparisons of the variant allele frequencies (VAFs) of the top mutated genes revealed that *TP53*, *PIK3CA*, *PTEN*, and *RB1* were the top 4 VAFs, with absolute high VAFs of greater than 30% (Supplemental Figure 2A). The mutation site with the highest frequency was found to be *PIK3CA* p.H1047R, accounting for 12% of the observed mutations (Figure 2B). In our mTNBC cohort, *MYC* was the most frequently copy number altered gene; it occurred in 54% of patients. The vast majority of these alterations were amplifications (Figure 2C).

To investigate the interrelationship between genomic alterations, we analyzed the co-occurrence and exclusivity of mutated genes. We did not observe any significant mutual exclusivity but identified several co-occurring patterns among these mutant genes. Among them, there were extensive co-occurring mutations in *PKD1* and *KMT2B* (Supplemental Figure 2B). In addition, the chromatin histone modifier pathway presented the highest frequency of co-occurring mutations with other pathways (Supplemental Figure 2C). Overall, our study reveals the largest comprehensive genomic landscape of TNBC metastases to date, contributing to a deeper understanding of TNBC metastases.

### Multidimensional comparative analysis of the genomic landscape underscores its uniqueness.

To investigate the organotropism of specific genomic signatures in TNBC metastases, we compared genomic alterations across various metastatic organs. The oncoplot shows the genomic features of specific organs grouped by metastases (Supplemental Figure 2D). We observed notable variations in the distribution of *PIK3CA* mutations among different organs (Supplemental Figure 2E, *P* < 0.05). Notably, the lesions metastasizing to the breast exhibited a higher prevalence of *PIK3CA* mutations compared with metastases in other organs. The biological mechanism behind this phenomenon deserves further study.

We next compared the differences in genomic mutations between Chinese and Western populations. The MSKCC mTNBC cohort (33), which includes publicly available data regarding TNBC metastases, was included as a representation of the Western mTNBC cohort. The most prevalent somatic mutation of TNBC metastases in the MSKCC cohort was that of *TP53* (93%), followed by *PIK3CA* (13%) and *NF1* (11%) (Supplemental Figure 3A). Compared with the Chinese FUSCC mTNBC cohort, the MSKCC cohort presented higher mutation rates in *TP53* (93% vs. 76%), *NF1* (11% vs. 4%), *BRCA1* (6% vs. 1%), and *FGFR4* (4% vs. 1%) but lower mutation rates in *PIK3CA* (13% vs. 21%), *CREBBP* (1% vs. 6%), and *KMT2B* (1% vs. 6%) (Supplemental Figure 3B). Compared with Western patients, Chinese patients with mTNBC presented significantly fewer alterations in genome integrity (82% vs. 94%) and cell cycle pathways (8% vs. 16%) (Supplemental Figure 3C).

**Figure 3:**
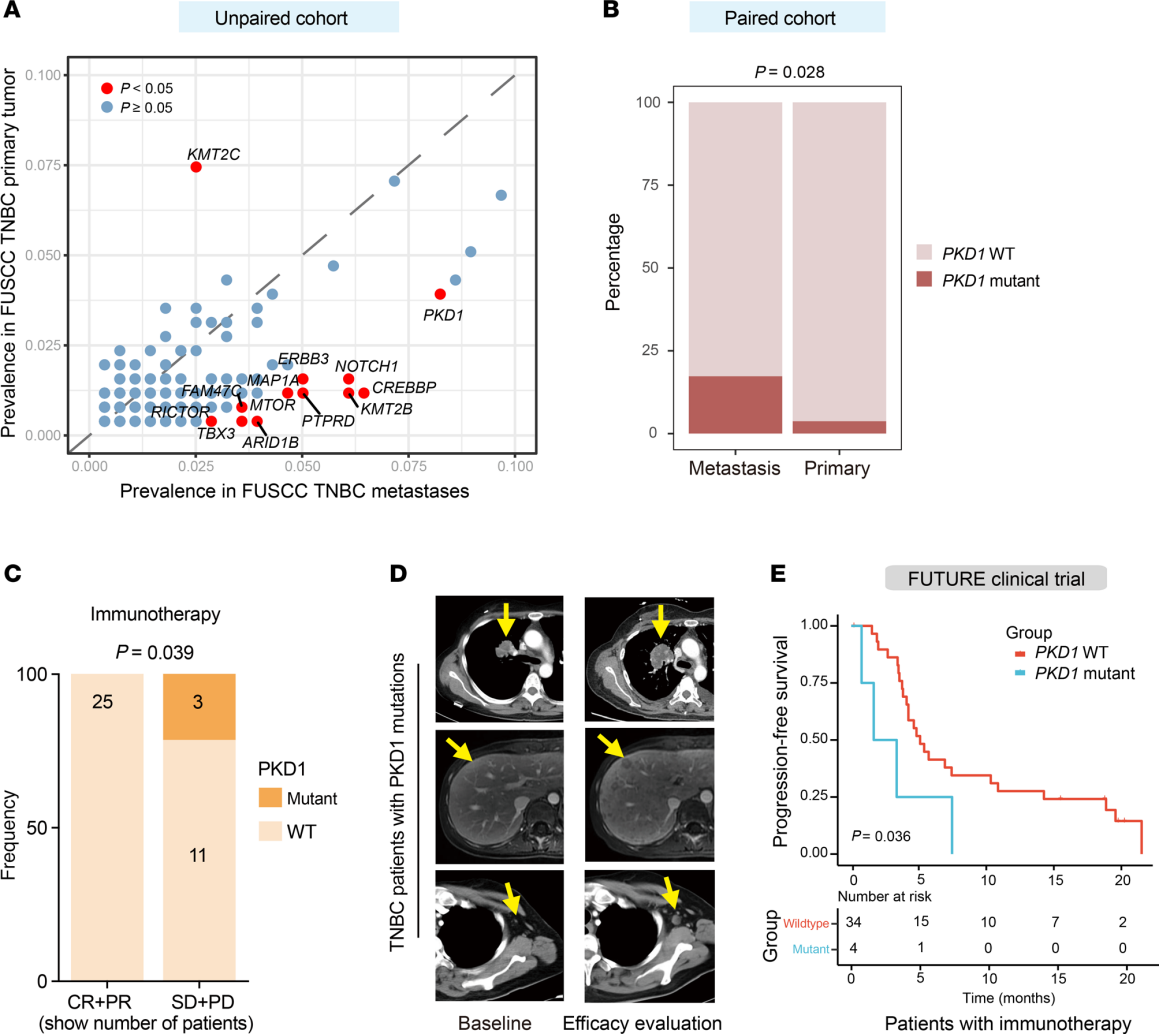
Genomic profiling of TNBC metastases identifies PKD1 as a potential biomarker for immunotherapy. (**A**) Comparative analysis of genomic mutations between large-scale unpaired metastatic (*n* = 296) and primary (*n* = 252) TNBC cohorts. *P* values were calculated via the χ^2^ test. (**B**) Validation of *PKD1* mutation enrichment in metastases using a paired cohort of 52 metastatic and 53 primary TNBC samples. *P* values were calculated using Fisher’s exact test. (**C**) Association between the objective response (CR+PR) rate and *PKD1* mutation status of immunotherapy in the FUSCC mTNBC cohort. Treatment responses were assessed according to RECIST v1.1 and systematically documented in electronic medical records based on radiographic evaluations. *P* values were calculated via the χ^2^ test. (**D**) Typical imaging of tissues from patients with advanced TNBC with *PKD1* mutations before and after immunotherapy. Yellow arrows indicate tumor lesions at baseline (left panels) and post-treatment (right panels). (**E**) Progression-free survival of patients with advanced TNBC receiving immunotherapy in the FUTURE trial (NCT03805399). *P* values were calculated via the log-rank test. TNBC, triple-negative breast cancer; FUSCC, Fudan University Shanghai Cancer Center; mTNBC, metastatic triple-negative breast cancer; WT, wild-type; RECIST v1.1, Response Evaluation Criteria in Solid Tumors version 1.1; CR, complete response; PR, partial response; SD, stable disease; PD, progressive disease.

The genomic data from the established TNBC metastases cohort was compared with the primary TNBC data from our center (8, 32) to identify novel genomic alterations that may contribute to tumor progression. Compared with primary tumors, many gene mutations, such as *PKD1*, *MTOR, NOTCH1* and *ERBB3*, were enriched in the metastatic lesions, whereas only that of *KMT2C* was more highly enriched in the primary lesions (Figure 3A). We further analyzed a new cohort of 105 tumor samples from 52 patients with primary and metastatic TNBC (including 1 case with 2 primary tumors matched to 1 metastatic site). Mapping the mutational landscapes of these matched pairs confirmed the findings in the above unpaired cohort: *PKD1* mutations were significantly enriched in metastatic lesions compared with their matched primary tumors (Figure 3B). These findings are consistent with the frequent implication of *MTOR*, *NOTCH1*, and *ERBB3* signaling in tumor progression and therapy resistance (34–38), lending credibility to our comparisons and highlighting the need for further investigation of the potential influence of PKD1 on malignant tumor behavior.

To explore the factors contributing to disparities in genetic mutation profiles between metastases and primary lesions (18, 39), we specifically investigated the influence of treatment pressure and natural disease progression. First, we compared the mutation frequency between treatment-naive TNBC metastases and primary lesions and discovered that metastases were enriched in expression of a significant number of genes (such as *NOTCH1* and *ERBB3*, Supplemental Figure 4A). Next, we conducted an internal comparison of metastases under different treatment pressures. Our results revealed that treatment-naive and pretreated metastases still presented distinct genomic differences (Supplemental Figure 4B). These findings suggest that primary and mTNBC lesions present significant differences in genomic mutations, which are likely influenced by treatment pressure and the natural progression of the disease. Owing to the lack of detailed neoadjuvant and adjuvant treatment histories, residual confounding cannot be ruled out. Therefore, further validation in prospective, well-annotated cohorts is necessary.

### Efficacy analysis reveals PKD1 expression as a potential biomarker for immunotherapy.

After biopsy, patients received real-world, guideline-directed systemic therapy (Supplemental Figure 5A). Given the expanding use of immune-checkpoint inhibitors in mTNBC, we performed a genomics-based analysis of immunotherapy efficacy. In our FUSCC mTNBC cohort, 39 patients received anti–PD-1 immunotherapy and had a full response assessment. Our genomic analysis revealed an intriguing correlation between polycystin-1 (PKD1) and immunotherapy resistance. Among the 3 patients with *PKD1* mutations, all experienced disease progression during the initial efficacy assessment according to Response Evaluation Criteria in Solid Tumors version 1.1 (RECIST v1.1). In contrast, the objective response rate to immunotherapy in the *PKD1*-WT group was 69.4% (25 of 36, *P* = 0.039; Figure 3C). The efficacy assessments and detailed clinical outcomes of these 3 patients before and after immunotherapy are shown in Figure 3D.

Based on the previously mentioned findings, we investigated the relationship between *PKD1* mutations and the efficacy of immunotherapy in 3 independent clinical trials in patients with advanced TNBC conducted at our center: the FUTURE trial (NCT03805399), the FUTURE-C-PLUS trial (NCT04129996), and the FUTURE-SUPER trial (NCT04395989). Remarkably, our findings consistently demonstrated that *PKD1* mutations were potentially associated with diminished immunotherapy response in TNBC (Figure 3E and Supplemental Figure 5, B and C). However, given the small clinical cohort size, these results should be interpreted with caution and warrant validation in larger prospective cohorts. Overall, we demonstrate that genomic profiling of metastases identified *PKD1* as a potential biomarker for immunotherapy.

### A multiomics study revealed the association of PKD1 with a “desert” tumor immune microenvironment.

Upon investigation, we discovered that the *PKD1* mutation rate in metastases was as high as 8% (the specific mutation sites are shown in Supplemental Figure 6), ranking sixth among all mutated genes in TNBC metastases (Figure 2A). Furthermore, *PKD1* mutations were more prevalent in TNBC metastases than in primary tumors (Figure 3, A and B), indicating that PKD1 may play an important role in TNBC progression and immune escape. More importantly, patients with *PKD1* mutations were resistant to immunotherapy (Figure 3E and Supplemental Figure 5, B and C). Based on the findings mentioned above, we investigated the influence of *PKD1* expression on TNBC, as few studies have explored this effect.

**Figure 4:**
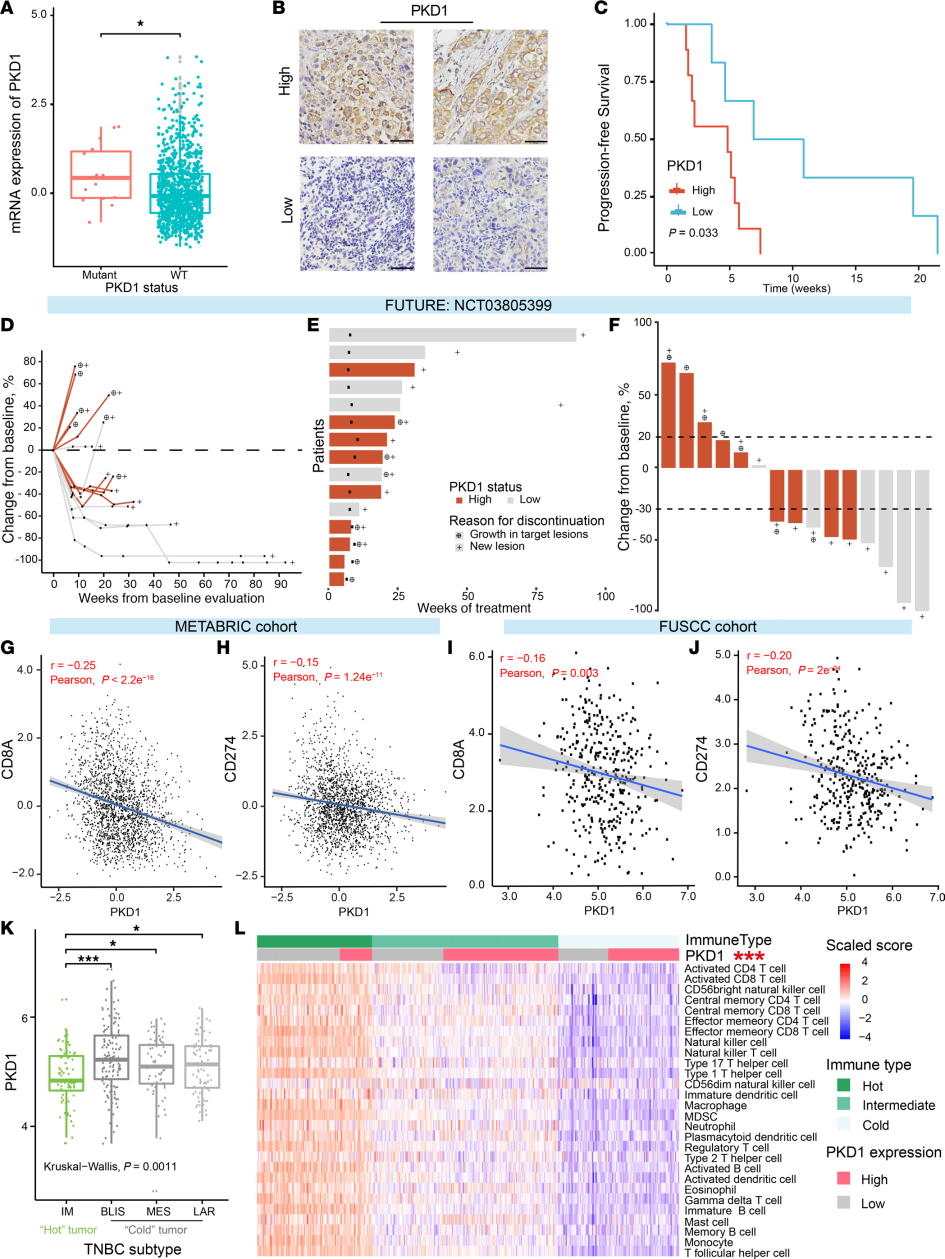
PKD1 is associated with a “desert” tumor immune microenvironment. (**A**) Comparative analysis of PKD1 mutant and WT mRNA expression in TCGA data. *P* values were calculated via the Wilcoxon test. (**B**) Representative immunohistochemical images of high and low PKD1 expression. Scale bar: 50 μm. (**C**) Progression-free survival with immunotherapy in patients with TNBC stratified by PKD1 expression levels in the FUTURE trial. A total of 16 patients who received immunotherapy in the FUTURE trial had sufficient pathological slices for PKD1 expression staining assessment. *P* values were calculated via the log-rank test. (**D**) Changes in the sum of target lesion diameters relative to baseline in patients with differential PKD1 expression in the FUTURE trial. Among the 16 patients, 1 was excluded because of a lack of posttreatment target lesion assessment data. (**E**) Duration of immunotherapy in patients with different PKD1 expression levels. (**F**) Best percentage change relative to baseline in the sum of target lesion diameters. The dashed lines at +20% and –30% represent the thresholds for disease progression and partial response. (**G**–**J**) Correlations of PKD1 expression with CD8A (**G** and **I**) and CD274 (**H** and **J**) in the METABRIC (**G** and **H**) and FUSCC (**I** and **J**) databases. *P* values were calculated via Pearson’s correlation test. (**K**) PKD1 mRNA expression across TNBC molecular subtypes in the FUSCC database. *P* values were calculated via the Wilcoxon test and the Kruskal-Wallis test. (**L**) Heatmap depicting immune cell infiltration and its correlation with PKD1 expression in the tumor immune microenvironment of TNBC. *P* values were calculated via the χ^2^ test. TNBC, triple-negative breast cancer; FUSCC, Fudan University Shanghai Cancer Center; IM, immunomodulatory; LAR, luminal androgen receptor; BLIS, basal-like immune-suppressed; MES, mesenchymal-like. *P < 0.05; **P < 0.01; ***P < 0.001.

Our subsequent analysis of multiomics data from The Cancer Genome Atlas (TCGA) databases further confirmed a correlation between *PKD1* mutations and increased mRNA expression (Figure 4A). Moreover, we extended our study to survival data of patients with TNBC (*n* = 360) in our multi-omics cohort (8) and found a potential association between high expression of PKD1 and poor prognosis in patients with TNBC (Supplemental Figure 7), although these observations require validation in prospective cohorts.

We next assessed immunohistochemical staining for PKD1 in patients undergoing anti–PD-1 immunotherapy in the FUTURE clinical trial (NCT03805399, Figure 4B). Further analysis revealed that patients with high PKD1 expression had poorer progression-free survival under immunotherapy (Figure 4C). Specifically, in this cohort, patients with high PKD1 expression presented characteristics such as increased tumor proliferation, a shorter duration of treatment, and less pronounced tumor regression in response to immunotherapy (Figure 4, D–F), warranting further investigation into its molecular mechanisms.

Considering the previously revealed effect of PKD1 expression on immunotherapy and disease prognosis, we conducted a comprehensive analysis of the impact of PKD1 expression on the tumor immune microenvironment (TIME). Initially, we focused on 2 commonly used clinical biomarkers for the immunotherapy response: CD8 (encoded by the CD8A gene) and PD-L1 (encoded by the CD274 gene). We observed a consistent negative correlation between PKD1 expression and that of each biomarker in our multiomics TNBC cohort (FUSCC) and in the METABRIC cohort (European Genome-phenome Archive, EGAS00000000083; https://ega-archive.org/studies/EGAS00000000083) (Figure 4, G–J), indicating that PKD1 may potentially serve as a biomarker for immunotherapy and requires further exploration and validation. Furthermore, we analyzed the expression of PKD1 in the 4 molecular subtypes of TNBC and found that its expression was significantly lower in the immunomodulatory (IM) subtype than in the other 3 subtypes (Figure 4K). Subsequent gene set enrichment analysis (GSEA) (40) revealed that patients with TNBC with *PKD1* mutations were enriched with numerous immune-negative Treg signaling pathways (Supplemental Figure 8). Additionally, we employed single-sample GSEA (41), which indicated that patients with TNBC with high PKD1 expression exhibited limited immune cell infiltration within the TIME, suggesting a “desert” TIME (Figure 4L).

### PKD1 promotes tumor immune evasion.

**Figure 5:**
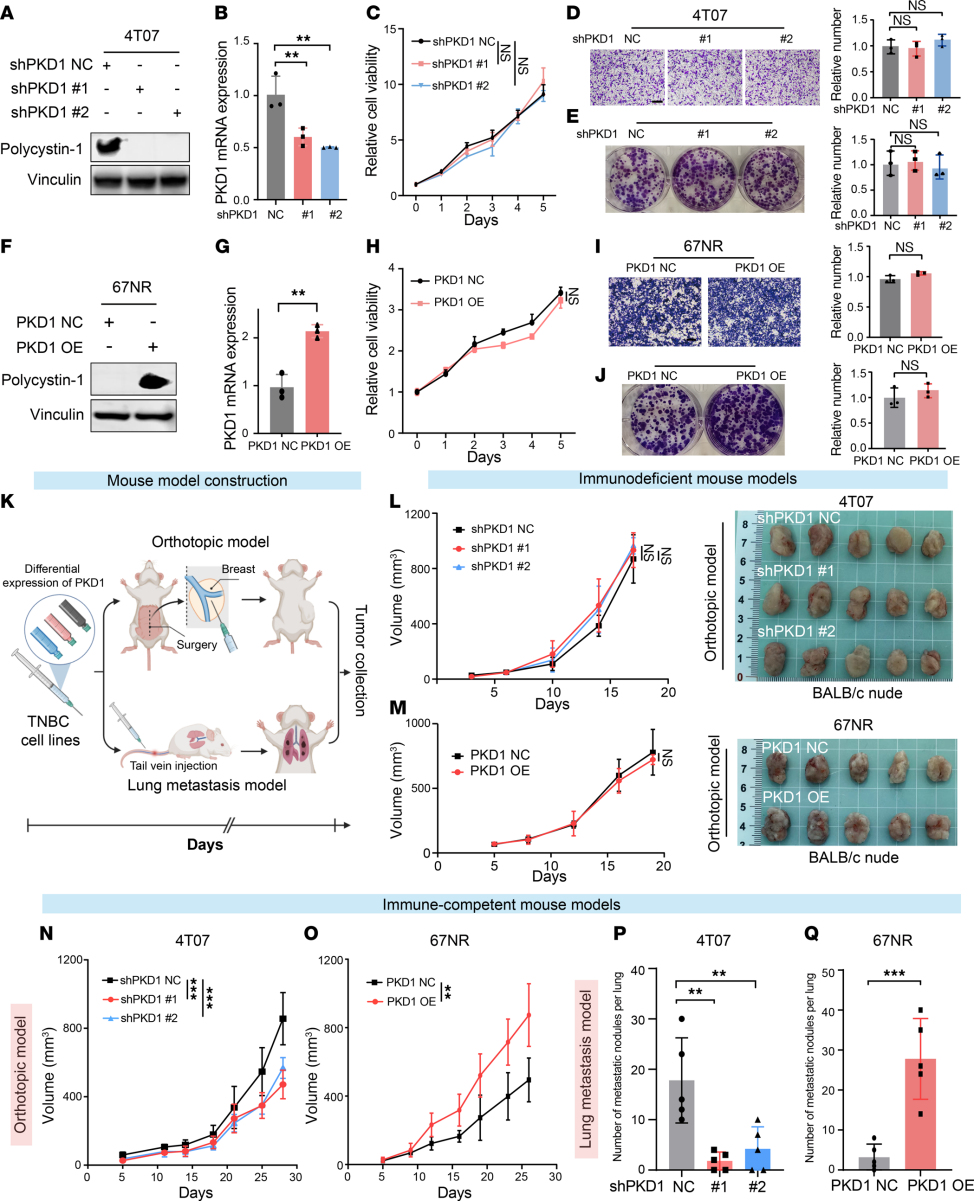
PKD1 expression facilitates tumor immune evasion. (**A** and **B**) PKD1 knockdown efficiency in 4T07 cells was validated by Western blotting (**A**; polycystin-1 protein) and qRT-PCR (**B**; PKD1 mRNA). (**C**) Growth curves of 4T07 cells in the PKD1-KD and NC groups. (**D** and **E**) Transwell migration (**D**) and clonogenic assays (**E**) of 4T07 cells in the PKD1-KD and NC groups. Representative images (left) and quantitation (right) are shown. Scale bar: 200 μm. (**F** and **G**) PKD1 overexpression efficiency in 67NR cells, analyzed by Western blotting (**F**) and qRT-PCR (**G**). (**H**) Growth curves of 67NR cells in the PKD1-OE and NC groups. (**I** and **J**) Transwell migration (**I**) and clonogenic assays (**J**) of 67NR cells in the PKD1-OE and NC groups. Representative images (left) and quantitation (right) are shown. Scale bar: 200 μm. (**K**) Schematic of the TNBC orthotopic tumor model and lung metastasis model construction. (**L** and **M**) Tumor growth in 4T07 (**L**) and 67NR (**M**) orthotopic allografts established in immunodeficient mice. (**N** and **O**) Tumor growth curves of the 4T07 (**N**) and 67NR (**O**) orthotopic tumor models in immunocompetent mice. (**P**) Number of lung metastases in immunocompetent mice bearing 4T07 tumors. (**Q**) Number of lung metastases in immunocompetent mice bearing 67NR tumors. Dunnett’s test was used in **B**–**E**, **L**, **N** and **P**. Two-tailed unpaired Student’s *t* test was used in **G**–**J**, **M**, **O** and **Q**. NC, negative control; KD, knockdown; OE, overexpression; TNBC, triple-negative breast cancer. *P < 0.05; **P < 0.01; ***P < 0.001.

We conducted basic translational research to investigate the underlying mechanisms by which PKD1 expression influences TNBC outcomes and the TIME. We assessed PKD1 expression levels among murine TNBC cell lines via both Western blotting (WB) and quantitative reverse transcription–PCR (RT–qPCR) and generated TNBC cell lines exhibiting differential PKD1 expression: 4T07 and TS/A were subjected to shRNA-mediated expression knockdown (42), whereas 67NR and 168FARN were overexpressed via the dCas9-SAM system (43) (Figure 5, A–J, and Supplemental Figure 9, A–F). We performed a series of in vitro experiments on the 4 aforementioned cell lines and observed that modulating PKD1 expression did not significantly affect tumor proliferation or migration in vitro (Figure 5, A–J, and Supplemental Figure 9, G–L).

To assess the effect of PKD1 expression on tumor growth in vivo, we utilized cell lines with differential PKD1 expression to establish various models in BALB/c mice: lung metastasis models and orthotopic tumor models, immunodeficient mouse models, and immune-competent mouse models (Figure 5K). Interestingly, in immunodeficient mouse models, differential PKD1 expression did not affect tumor growth (Figure 5, L and M). However, in immune-competent mice, PKD1 expression significantly influenced tumor growth (Figure 5, N–Q). Specifically, in the orthotopic tumor model, tumor growth was markedly suppressed in the PKD1-knockdown (PKD1-KD) group compared with the control group (Figure 5N). Similarly, in the lung metastasis model, compared with the control group, the PKD1-KD group presented a significant reduction in the number of lung metastases (Figure 5P). Consistent results were observed in models subjected to PKD1 overexpression, in which PKD1 expression promoted tumor proliferation and lung metastasis (Figure 5, O and Q).

On the basis of the experimental results from in vitro and in vivo models, as well as conclusions drawn from clinical cohorts regarding PKD1-mediated immunotherapy resistance, we hypothesized that PKD1 expression may play an important role in immune evasion.

### Targeting CCL2 overcomes PKD1-mediated immunotherapy resistance.

**Figure 6:**
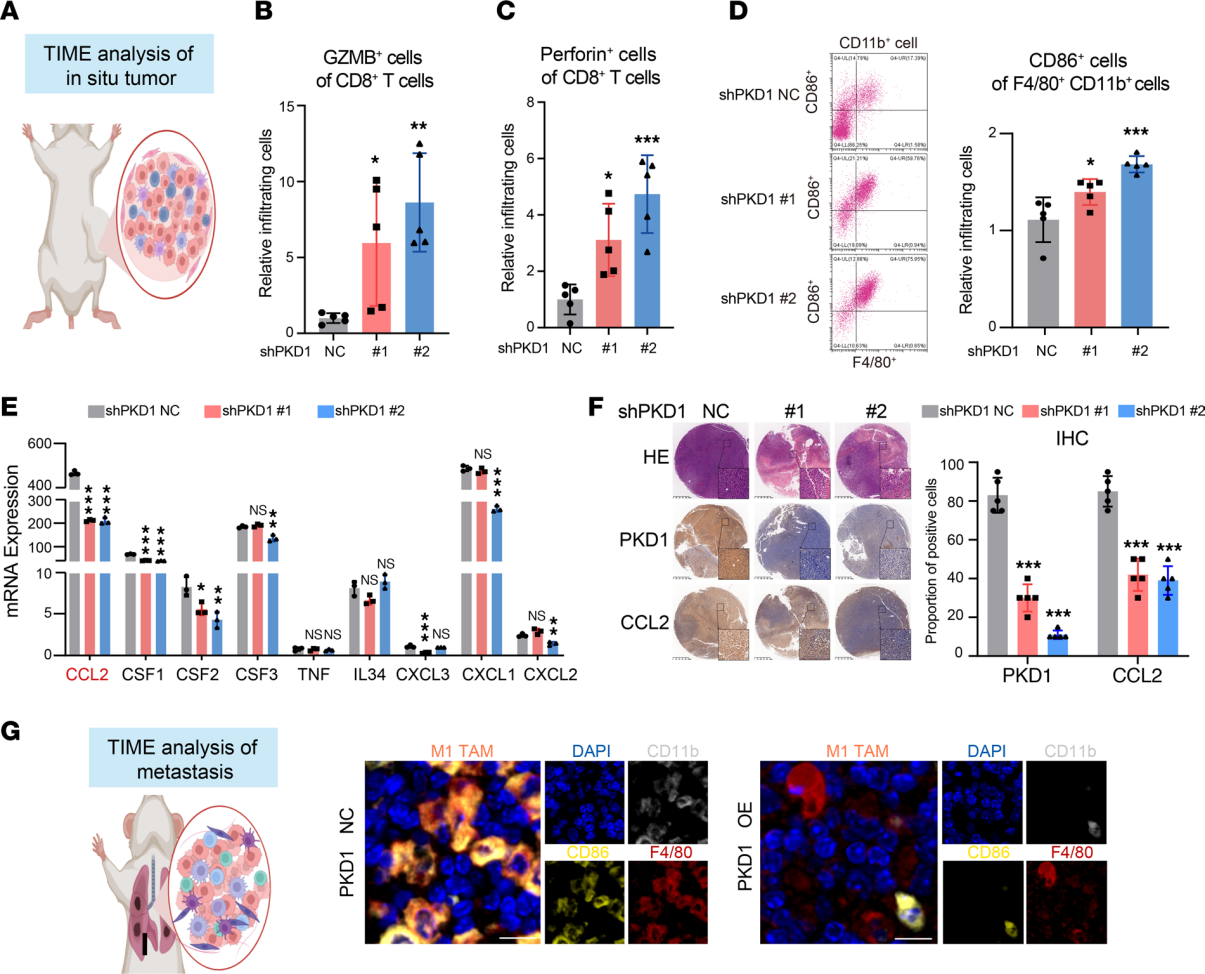
PKD1 promotes an immunosuppressive tumor microenvironment in TNBC. (**A**) Schematic of TIME analysis of TNBC orthotopic tumors. (**B** and **C**) The infiltration levels of GZMB^+^ cells (**B**) and perforin^+^ cells (**C**) among CD8^+^ cells in the TIME analysis of 4T07 allograft orthotopic tumors were detected by flow cytometry. (**D**) The infiltration levels of CD11b^+^CD86^+^F4/80^+^ cells (M1-type TAMs) in the TIME analysis of 4T07 allografted orthotopic tumors. Left: Representative flow cytometric images illustrating the proportion of M1-type TAMs. Right: The relative quantification results of infiltrating cells in the PKD1-KD and NC groups. (**E**) mRNA expression of macrophage-related chemokines and cytokines in the PKD1-KD and NC groups. (**F**) Immunohistochemistry showing the expression of PKD1 and CCL2 in 4T07 allograft orthotopic tumor tissue microarrays Scale bars: 125 μm (main panels), 10 μm (insets). The expression of PKD1 and CCL2 is quantified on the right. (**G**) TIME analysis of TNBC metastasis. Multiplex immunofluorescence staining for M1-type TAMs in the lung metastases of mice from the PKD1-NC and OE groups. Dunnett’s test was used in **B**–**F**. TIME, tumor immune microenvironment; TNBC, triple-negative breast cancer; TAMs, tumor-associated macrophages; NC, negative control; KD, knockdown; OE, overexpression. *P < 0.05; **P < 0.01; ***P < 0.001.

To elucidate the precise effect of PKD1 expression on tumor immune evasion, we performed a series of analyses on the TIME associated with the differential expression of PKD1. Flow cytometry analysis of TIME cells in situ revealed significant differences in infiltrating immune cells between the groups (Figure 6, A–D). In the PKD1-KD group, the proportions of GZMB^+^ and perforin^+^ CD8^+^ T cells increased significantly (Figure 6, B and C). In addition, knockdown of PKD1 expression significantly increased the infiltration level of M1-type tumor-associated macrophages (TAMs) in situ, which facilitate antitumor immune activation (Figure 6D). RNA-seq analysis revealed that the low-expression PKD1 group exhibited significant enrichment in multiple macrophage-related pathways, including cytokine activity, chemokine activity and the C-C motif chemokine receptor 2 (CCR2) signaling pathway (Supplemental Figure 10). Combining the phenomenon of TAM reprogramming and the large number of enriched chemokine- and cytokine-related pathways demonstrated by RNA-seq analysis, we analyzed chemokines and cytokines that could affect TAM infiltration (44, 45) and found that the expression of C-C motif chemokine ligand 2 (CCL2) was significantly decreased after PKD1 expression knockdown (Figure 6E). As demonstrated by immunohistochemistry (IHC) of tumor tissues, the expression of CCL2 significantly decreased after PKD1 expression knockdown (*P* < 0.05, Figure 6F). Multiplex immunohistochemical analysis of the TIME in metastatic lesions further demonstrated that PKD1 expression suppressed the infiltration of M1-type TAMs (Figure 6G).

Previous studies have reported that CCL2 is an important macrophage chemokine that inhibits the infiltration of M1 macrophages (44, 45). Taken together, our findings suggest that PKD1 might mediate immune evasion in TNBC by upregulating CCL2 to inhibit M1-type TAM infiltration, thus mediating a desert TIME.

**Figure 7:**
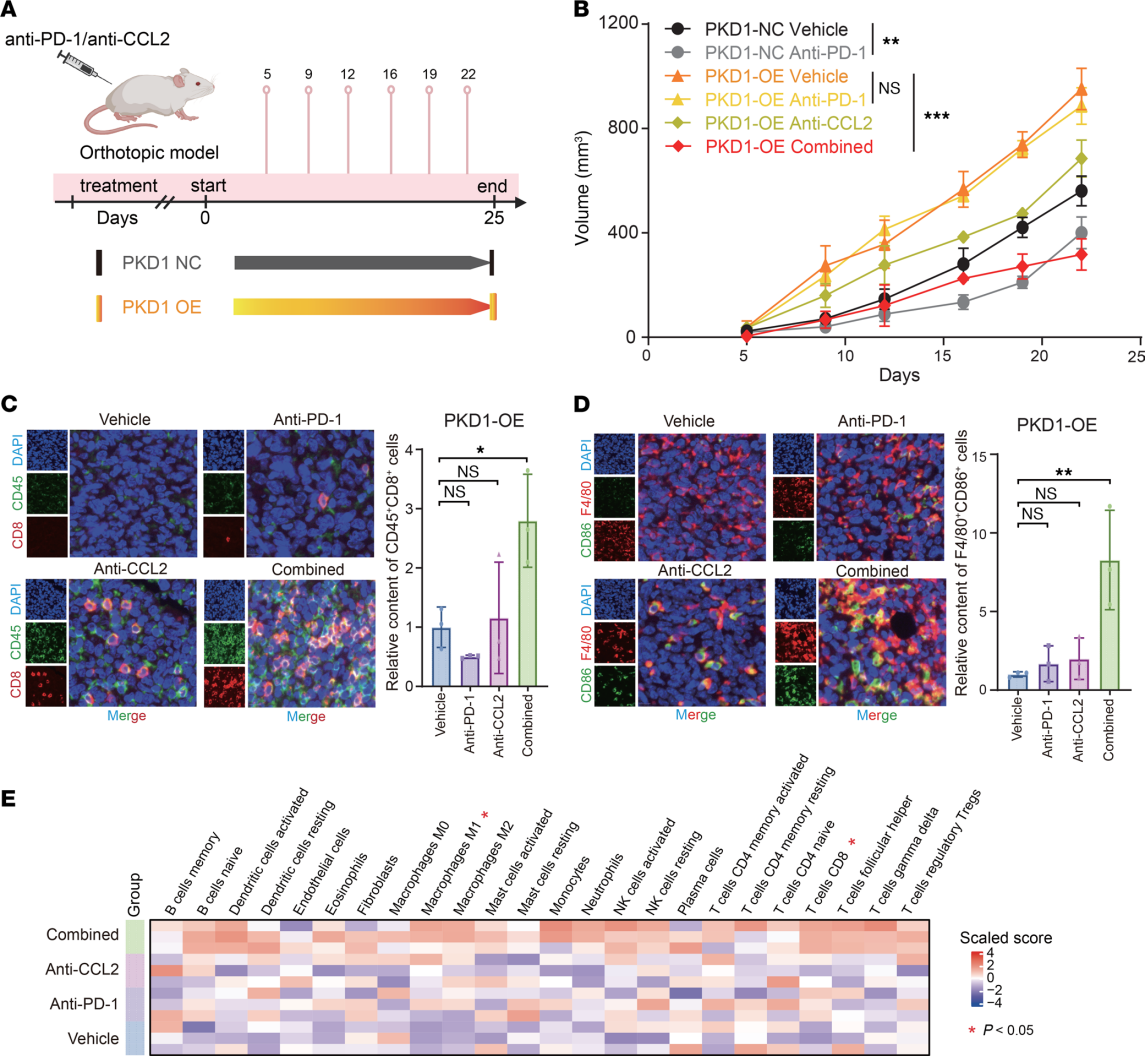
Targeting CCL2 overcomes PKD1-mediated immunotherapy resistance. (**A**) Schematic of the treatment with anti–PD-1 and/or anti-CCL2 in mice from the PKD1-NC and PKD1-OE groups. (**B**) Tumor growth curves of mice in the PKD1-NC and PKD1-OE groups treated with anti–PD-1 and/or anti-CCL2 antibodies. *P* values were calculated via 2-tailed unpaired Student’s *t* tests. (**C**) Multiplex immunofluorescence results of CD45^+^CD8^+^cells. Left: Representative multiplex immunofluorescence images. Right: Quantitative analysis across treatment groups. Statistical comparisons were performed using Dunnett’s test, with the vehicle group designated as the reference control. (**D**) Multiplex immunofluorescence results of F4/80^+^CD86^+^cells. Left: Representative multiplex immunofluorescence images. Right: Quantitative analysis across treatment groups. *P* values were assessed using Dunnett’s test. (**E**) Heatmap showing immune cell infiltration in the tumor immune microenvironment across treatment groups (vehicle, anti–PD-1, anti-CCL2, and combined), based on bulk RNA-seq of murine TNBC. *P* values were calculated using the Kruskal-Wallis test. CCL2, C-C motif chemokine ligand 2; TNBC, triple-negative breast cancer; NC, negative control; OE, overexpression. *P < 0.05; **P < 0.01; ***P < 0.001.

Given a lack of drugs that directly target PKD1, we explored new treatment strategies to counteract the PKD1-mediated desert TIME and immunotherapy resistance. In an immune-competent orthotopic tumor model overexpressing PKD1 (Figure 7A), we reconfirmed that PKD1 expression promoted tumor growth and mediated resistance to anti–PD-1 therapy. Notably, the combination of anti–PD-1 and anti-CCL2 treatments significantly inhibited the growth of tumors overexpressing PKD1 (Figure 7B). To assess changes in the TIME after treatment, we performed multiplex immunofluorescence (Figure 7, C and D) and bulk RNA-seq (Figure 7E) on tumor tissues collected after the final antibody administration. The results indicated that the combination treatment group exhibited overall higher immune cell infiltration and a more immunologically active microenvironment compared with monotherapy groups, along with significant enrichment of M1-type TAMs and CD8^+^ T cells (Figure 7, C–E). These findings demonstrate that targeting CCL2 can overcome PKD1-mediated immunotherapy resistance, suggesting a potential therapeutic option for addressing immunotherapy resistance in patients with TNBC.

## Discussion

Genomic profiling of mTNBC tissues is crucial for advancing precision treatment strategies. While primary TNBC has been extensively studied, the genomic alterations specific to metastases, particularly in Asian populations, remain underexplored. In our study, large-scale DNA sequencing provided a comprehensive genomic characterization of TNBC metastases and advanced precision treatment strategies for mTNBC. By integrating sequencing data with efficacy analyses, we identified PKD1 as a key modulator of immune evasion and proposed a potential strategy to overcome immunotherapy resistance.

Our study integrates two complementary designs: a large, prospectively accrued unpaired metastatic cohort (*n* = 296) and a rigorously matched primary-metastatic paired cohort (*n* = 105). The large unpaired mTNBC cohort provided statistical power to identify metastatic-enriched genomic alterations across a diverse patient population and captured the breadth of genomic heterogeneity encountered in routine clinical practice, yet it precludes patient-level evolutionary inference. Conversely, the paired mTNBC cohort allowed direct tracing of clonal selection and confirmed that *PKD1* mutations are significantly enriched in metastases relative to their matched primaries, validating observations from the unpaired series. Nevertheless, both cohorts share limitations, including potential sampling bias; the use of panel sequencing, which restricts the scope of genomic alterations; and a lack of multiregion sampling to fully resolve intratumoral heterogeneity. In particular, the application of laser capture microdissection or spatial genomics will be critical to address the challenge of intratumoral spatial heterogeneity. Future prospective studies incorporating multi-region sampling of primary and metastatic sites, complemented by whole-genome or whole-exome sequencing, will be essential to fully elucidate the evolutionary history of mTNBC.

Our study reinforces previous reports regarding the importance of genomic research in comprehending disease onset, progression, and personalized precision therapies (9, 46, 47). Notably, through a well-defined and large-scale cohort, we offer distinct contributions to this field. First, our cohort is primarily composed of Asian patients, filling the previous knowledge gap regarding the genetic landscape of mTNBC in the Asian population (19, 26, 30, 31, 33). However, the comparison between Asian and mixed European cohorts, while suggesting potential population-specific differences, must be interpreted with caution due to inherent limitations of retrospective designs. Differences in treatment protocols, dosing strategies, baseline pathological features (e.g., histological grading), and unmeasured genetic variations (e.g., pharmacogenomic single-nucleotide polymorphisms, human leukocyte antigen alleles) may confound observed disparities. Owing to missing clinical variables in the publicly available dataset, we were unable to perform comprehensive multivariable adjustment. Baseline imbalances may confound direct cross-cohort comparisons. Future prospective, multicenter studies with standardized treatment protocols and comprehensive genetic profiling are needed to definitively address cross-population differences in TNBC biology and treatment response.

We focused on mTNBC, the most advanced and aggressive stage of breast cancer (2, 48). Once TNBC disseminates distally, therapeutic challenges become exceedingly intricate. These challenges predominantly involve the pronounced heterogeneity of TNBC, which limits the use of targeted therapies, the constrained efficacy of conventional chemotherapy, and the variable effectiveness and tolerability of immunotherapies (49–51). Previous studies have focused primarily on molecular subtyping and potential therapeutic targets in early-stage TNBC (52, 53). To provide a more comprehensive understanding of the genomic evolution in metastases of TNBC, we have integrated our findings with previous reports demonstrating substantial geno- and phenotypic differences between primary tumors and metastases (18, 20, 26, 33, 54). Numerous studies have highlighted receptor status changes (16) and acquisition of new mutations (55, 56) during disease progression, which have important implications for treatment decision-making. Discordant mutation profiles between primary tumors and distant metastases are increasingly recognized as hallmarks of tumor evolution (18–20). Our genomic analyses revealed both the acquisition of new mutations (e.g., in *PKD1*, *MTOR*, *NOTCH1*) and the apparent loss of certain alterations (e.g., in *KMT2C*) present in primary tumors during metastatic progression. Several mechanistic explanations may account for this divergence: (a) clonal selection under metastatic pressure can enrich subclones carrying driver mutations that enhance dissemination or immune evasion; (b) tumor purity and sequencing depth may limit detection of low-abundance clones; (c) metastatic-site-specific microenvironmental pressures (hypoxia, immune surveillance) may further sculpt the genomic landscape. The convergence of our findings with previous studies suggests fundamental biological principles governing metastatic evolution. These observations support international guidelines that recommend metastatic biopsies for accurate classification, exclusion of alternative diagnoses, and improved treatment planning (17). Our study builds on these insights by offering large-scale, metastatic tissue-based genomic data from patients with TNBC, further elucidating the molecular heterogeneity that emerges in advanced disease (8, 57). Finally, our large TNBC metastatic cohort and genomic dataset of TNBC metastases could serve as a useful public resource to promote precision treatment of TNBC. As emphasized earlier, future studies incorporating multiregion sequencing of primary tumors and deep sequencing of matched metastases will help distinguish true biological selection from technical artifacts.

Immunotherapy has shown substantial benefits for a subset of patients with TNBC; however, a portion of patients is immunotherapy resistant, highlighting the urgent need for well-defined genomic biomarkers to guide treatment strategies (58, 59). The success of immune checkpoint blockade combined with chemotherapy in the primary TNBC setting, as demonstrated by the KEYNOTE-522 trial (10, 60–62), underscores the potential for effective antitumor immunity when the microenvironment is permissive. Our findings suggest that the metastatic niche may be actively reshaped by specific genomic alterations, such as *PKD1*, which was linked to a desert TIME, contributing to immune evasion and resistance to immunotherapy. This suggests that the immunosuppressive circuitry driven by alterations such as PKD1 may override the potential immunogenic benefit conferred by a high tumor mutational burden in metastases, thereby contributing to the attenuated efficacy of immunotherapy in advanced TNBC. However, these findings stem from retrospective analyses, and future (a) prospective randomized trials with standardized immune and germline profiling and (b) intrapatient paired biopsy/blood protocols are needed for deeper exploration and more robust validation. The *PKD1* gene is located on human chromosome 16p13 and encodes the transmembrane glycoprotein polycystin-1. Previous studies have shown that *PKD1* mutations are closely associated with autosomal dominant polycystic kidney disease (63, 64). The scarcity of research on PKD1 expression in breast cancer and immunotherapy highlights the importance of our findings, which reveal its potential roles in promoting immune evasion.

The importance of CCL2 in the TIME extends beyond its role in attracting macrophages to tumors; it also regulates the functions of M1-type and M2-type TAMs in antitumor immunity (65). M1-type TAMs are typically associated with immune activation and antitumor activity, whereas M2-type TAMs exhibit immunosuppressive properties that promote tumor progression. Previous studies have shown that CCL2 suppresses the function of M1-type TAMs and inhibits their CD8^+^ T cell–mediated antitumor activity, thereby exerting an immunosuppressive effect on the TIME (45, 65, 66). Therefore, balancing the influence of CCL2 on M1-type and M2-type TAMs is crucial for enhancing antitumor immune responses. In our study, we found that PKD1 expression inhibited M1-type TAM infiltration in the TIME through CCL2 signaling. Elucidating the specific mechanisms of the PKD1/CCL2/TAM regulatory axis is essential for guiding immunotherapy strategies. PKD1 can serve as a biomarker to precisely identify patients with TNBC who may benefit from immunotherapy. Conversely, combining anti-CCL2 therapy with anti–PD-1 treatment, which targets the PKD1/CCL2/TAM regulatory axis, may offer new therapeutic options for patients with TNBC with high PKD1 expression or *PKD1* mutations. Prospective clinical cohorts, augmented multi-omics datasets (e.g., single-cell sequencing, spatial proteomics), and deeper mechanistic investigations will be essential to fully clarify PKD1’s role in tumor immunology.

Moreover, the present study has several limitations. Although our analysis incorporated multidimensional genomic comparisons, potential residual confounding factors — such as differences in treatment protocols, dosing strategies, treatment history (including neoadjuvant and adjuvant regimens), and unmeasured genetic variations — cannot be excluded. Therefore, future multicenter studies with standardized treatment protocols and comprehensive genetic profiling are warranted for deeper mechanistic exploration and more robust clinical validation. Furthermore, owing to the relatively small cohorts of immunotherapy, the observed association between PKD1 and immunotherapy efficacy needs to be validated in larger prospective studies. Additionally, the high molecular weight of PKD1 presents a major challenge for directly constructing mutants. Nonetheless, we prioritized multidimensional analysis and foundational research regarding PKD1, highlighting its importance and potential role in mediating immunotherapy resistance in patients with mTNBC. Finally, mutations were not experimentally validated. To compensate, we applied stringent bioinformatic filters and manually inspected crucial variants to ensure the high confidence of our data.

In summary, our study provides a comprehensive genomic characterization of TNBC metastases, identifies PKD1 as a key modulator of immune evasion, and proposes a potential strategy to overcome immunotherapy resistance, contributing to the advancement of precision treatment for mTNBC.

## Methods

### Sex as a biological variable.

All human participants in our cohorts were female, which is representative of the patient population for breast cancer, a disease that predominantly affects females. For the in vivo studies, only female mice were used to establish orthotopic and metastatic tumor models.

### Study design and samples.

We prospectively collected 296 TNBC metastases and paired peripheral blood samples from patients with mTNBC treated at FUSCC from October 2018 to November 2020. Eligibility criteria included the following: (a) female patients diagnosed with metastatic breast carcinoma with an ER-negative, PR-negative, and HER2-negative phenotype by the Department of Pathology at FUSCC (the immunohistochemical cutoff for ER/PR-negative status was less than 1% staining in nuclei, and HER2-negative status was defined as a score of 0 or 1 by immunohistochemical analysis or the absence of ERBB2 amplification by fluorescence in situ hybridization with an IHC score); (b) metastatic samples were subjected to targeted NGS using the FUSCC-BC 484-gene panel (32); and (c) tumor and peripheral lymphocyte paired gene-panel reports were required to identify somatic genomic alterations.

The majority of metastatic samples were obtained via core needle biopsy under radiological guidance. For breast lesions, Mammotone Elite needles (gauge 10) were used; for lymph nodes, chest wall metastases and other sites, Bard needles (gauge 14) were employed. Tissue sampling was based on two key principles: (a) clinical safety of the biopsy procedure, and (b) ability to obtain sufficient tissue for both pathological diagnosis and genomic sequencing. No severe biopsy-related events were recorded in this study.

Clinicopathological characteristics included age; tumor histologic type; tumor size; lymph node status; histologic grade; and ER, PR, HER2, and Ki67 expression status. We also collected treatment and efficacy information in a real-world setting after tumor metastasis in patients. Lines of therapy were defined based on systemic treatments administered after the diagnosis of metastatic disease. In FUSCC, patient staging integrates findings from both imaging and surgical pathology. Clinical assessment of the disease is performed using MRI, CT, and PET-CT to screen for local lesions or distant metastases. Following surgery, the final pathological stage is assigned based on the histopathological examination of the resected specimens. The follow-up for this cohort of patients was completed on March 31, 2022. Overall survival was defined as the time from when NGS was performed on metastatic samples to death from any cause. Patients without events were censored from the time point of the last follow-up.

### Paired primary-mTNBC cohort.

Between April 2020 and May 2024, patients with mTNBC treated at FUSCC were enrolled. Eligibility criteria were as follows: (a) histopathological confirmation of TNBC in both the primary tumor and at least one matched distant metastatic lesion (ER/PR <1%, HER2 0–1+ or 2+ with negative FISH) and (b) successful completion of targeted NGS using the institutional FUSCC-BC gene panel on both specimens. All samples were reviewed by 2 independent breast pathologists to confirm histology and tumor content.

### TNBC multiomics patient cohort.

Patients who were treated at the FUSCC from January 2007 to December 2014 were enrolled according to the following defined criteria: (a) female patients diagnosed with unilateral invasive ductal carcinoma with an ER-negative, PR-negative, and HER2-negative status; (b) central pathologic examination of tumor specimens performed by the Department of Pathology at FUSCC; and (c) no evidence of distant metastasis at diagnosis. For more cohort information, please refer to our previous research report (8).

### FUTURE clinical trial cohort.

Between October 2018 and February 2022, patients who had heavily pretreated mTNBCs and had experienced disease progression during or following almost all standard chemotherapies were enrolled in the FUTURE trial (NCT03805399), a phase II umbrella trial. Patients with the IM subtype were assigned to arm C and received camrelizumab combined with chemotherapy. Thirty-eight patients in arm C with postbaseline tumor assessments of target lesions are represented in Figure 3E. For more cohort information, please refer to our previous research reports (67, 68).

### FUTURE-C-Plus clinical trial cohort.

Between October 2019 and October 2020, patients with previously untreated, advanced, IM TNBC were enrolled in the FUTURE-C-Plus trial (NCT04129996), an open-label, single-arm, phase II study. Eligible patients with TNBC received camrelizumab as first-line therapy. The data of 22 patients obtained via NGS and complete efficacy assessment are presented in Supplemental Figure 5B. For more cohort information, please refer to our previous research report (69).

### FUTURE-SUPER clinical trial cohort.

Between July 2020 and October 2022, patients with unresectable locally advanced or mTNBC at FUSCC were enrolled in the FUTURE-SUPER trial. This is a phase II, open-label, randomized controlled umbrella trial evaluating the efficacy and safety of multiple targeted treatments versus traditional chemotherapy in patients with mTNBC. Patients with the IM subtype were assigned to group C and received camrelizumab plus famitinib plus nab-paclitaxel. Thirteen patients in group C with NGS data and complete efficacy assessment data are represented in Supplemental Figure 5C. For more cohort information, please refer to our previous research report (70).

### Sequencing using the FUSCC-BC panel.

All specimens underwent pathological evaluation to confirm mTNBC diagnosis. Only those confirmed as mTNBC were included for genomic sequencing. Details on the sequencing protocol and FUSCC-BC panel have previously been described (32). All fresh frozen tumor specimens underwent pathological review. A board-certified breast pathologist examined H&E-stained sections derived from each sample to confirm the diagnosis and assess tumor cellularity. Only samples with a tumor cell content of 20% or greater were included for subsequent genomic analysis. Genomic DNA was coisolated from these qualified tumor tissues and matched peripheral blood mononuclear cells using the TGuide M24 system (Tiangen). The concentration and purity of the extracted DNA were determined using a NanoDrop 2000 spectrophotometer (Thermo Scientific), with an A260/A280 ratio between 1.6 and 1.9 being required for proceeding. Sequencing libraries were constructed from the genomic DNA using the KAPA HyperPlus kit (Kapa Biosystems) according to the manufacturer’s instructions. A custom-designed targeted gene panel, named the FUSCC-BC panel, was used for target enrichment. The enrichment was performed using in-house developed biotinylated RNA baits, which were transcribed from a synthesized oligo pool (Synbio Technologies). The final captured libraries were sequenced on the Illumina HiSeq X TEN platform (Illumina Inc.). The sequencing was performed to achieve a mean coverage depth of approximately 1,000× for tumor DNA. The resulting data were processed to identify single nucleotide variations, small insertions/deletions (indels), and CNVs. Key alterations were manually reviewed using the Integrative Genomics Viewer. This visual inspection was performed to confirm the actual presence of specific mutations in the patient’s aligned reads and to verify the consistency of the VAF, thereby validating the accuracy of the automated variant calling and eliminating artifacts or false positives.

### Cell culture and viability assay.

The murine TNBC cell lines (obtained from Y.B. Kang’s lab [Princeton University, Princeton, New Jersey, USA]) for functional studies were selected based on systematic Pkd1 expression profiling. 4T07, TS/A, 67NR, and 168FARN cells were maintained in RPMI 1640 medium (BasalMedia, L210) supplemented with 10% fetal bovine serum (Gibco, 10270–106) and 1% penicillin-streptomycin (BasalMedia, S110B), and the cells were not passaged more than 6 times from collection to use. The cells of interest (1 × 10^3^ to 3 × 10^3^ cells per well) were seeded into 96-well plates overnight in 100 μL complete growth medium in triplicate for the designated time. Cell viability was tested via a cell counting kit-8 assay (Dojindo Molecular Technologies, CK04) according to the manufacturer’s instructions.

### Expression vectors, plasmid transfection, and lentiviral infection.

Human *PKD1* short hairpin RNAs (shRNAs) in the U6-MCS-Ubiquitin-EGFP-IRES-puromycin vector (GV248) were purchased from GeneChem. Furthermore, given the substantial molecular weight of the PKD1 protein, we utilized the CRISPR/dCas9 synergistic activation mediator (SAM) system to overexpress PKD1 (71, 72). The human PKD1 CAS9-SAM plasmid was purchased from GeneChem. To generate stable cell lines expressing sgRNAs or shRNAs, each lentiviral expression vector was transfected into HEK293T cells with polyethyleneimine. The supernatant containing the viruses was collected 48 hours after transfection, filtered, and used to infect target cells in the presence of 10 μg/mL polybrene (Sigma-Aldrich, H9268) prior to drug selection with 1–2 μg/mL puromycin for 1 week. The overexpression (OE) and KD efficiencies were validated by immunoblotting after transfection.

### Real-time quantitative reverse transcription PCR.

Total RNA was isolated from cells via a FastPure Cell/Tissue Total RNA Isolation Kit V2 (Vazyme, RC112-01) following the manufacturer’s protocol. cDNA was synthesized via a HiScript Ill 1st Strand cDNA Synthesis Kit (+gDNA wiper) (Vazyme, R312-02). Real-time quantitative reverse transcription PCR (qRT-PCR) was performed in triplicate via ChamQ SYBR qPCR Master Mix (Vazyme, Q311-02) on an ABI 7900HT Fast Real-Time PCR System (Applied Biosystems). The results were analyzed via normalization to the GAPDH expression level via the 2^−ΔΔCT^ method.

### WB analysis.

The WB protocol has previously been described in detail (73). In brief, the cells were lysed in Pierce T-PER Tissue Protein Extraction Reagent (Thermo Fisher Scientific Inc.) containing protease and phosphatase inhibitors (Bimake, B14001, B15001A + B). The lysates were centrifuged at 13,500*g* for 15 minutes, the supernatants were collected, and the protein concentrations were determined with a Bicinchoninic (BCA) protein assay kit (Solarbio, PC0020). A total of 20–30 μg protein was separated by SDS-PAGE and transferred to PVDF membranes (Millipore, IPVH00010, ISEQ00010). The following primary antibodies were used: antiPKD1 (polycystin1; Santa Cruz Biotechnology, sc130554), antiGAPDH (Abcam, ab181602), and antiVinculin (Abcam, ab129002).

### RNA-seq analysis.

The methods used for RNA extraction, quality control, and sequencing have previously been described in detail (74). Total RNA from 4T07 cells was extracted via TRIzol Reagent (Invitrogen). The cDNA libraries created from the cell lines (3 samples in total, with 3 replicate libraries for each sample) were analyzed on a HiSeq 2500 instrument according to the manufacturer’s instructions (Illumina). GSEA was performed via the JAVA program using a specific gene set collection. One thousand random sample permutations were performed, and the significance threshold was set at the normalized enrichment score of absolute value >1, nominal *P* value < 0.05, and FDR *q* value < 0.05.

### Migration assay.

The migration assay has previously been described in detail (74, 75). A total of 5 × 10^4^ to 10 × 10^4^ cells were resuspended in the upper compartment of each chamber with 100 μL serum-free medium, and the lower compartment was filled with 600 μL medium containing 20% fetal bovine serum. The Transwell plates were incubated in a humidified environment with 95% air and 5% CO_2_ at 37 °C. The chambers were then washed with PBS, fixed with formaldehyde, and stained with 0.5% crystal violet.

### In vivo allograft model.

We employed TNBC cell lines to establish in vivo models (lung metastasis models, orthotopic tumor models, and spontaneous metastasis models) in 6-week-old female BALB/c mice (obtained from Shanghai Jihui Laboratory Animal Care Co., Ltd) weighing 15–16 g. We established a lung metastasis model by intravenously injecting 1 × 10^5^ TNBC cells in 100 μL PBS per mouse. For the orthotopic tumor model and spontaneous metastasis model, we injected 1 × 10^5^ TNBC cells in 10 μL PBS per mouse into the fourth mammary fat pad. Mice assigned to immunotherapy were given 5 intraperitoneal injections of 200 μg InVivoMAb anti-mouse PD-1 (Bio X Cell) or/and anti-mouse CCL2-InVivo (Selleck) every third day. Tumor volumes and the TIME profiles were utilized to evaluate immunotherapy response. Tumor volumes were monitored via caliper measurements every 3–4 days and were calculated as follows: length × width^2^/2. Euthanasia of the mice and collection of tumors were performed after the humane endpoint.

### IHC analysis.

Details on the IHC protocol have previously been described (75). For tissue microarray construction, 4T07 orthotopic tumors were harvested 21 days after implantation into BALB/c mice, as previously described (76, 77). Tumors were fixed in 10 % neutral-buffered formalin (24 h), paraffin-embedded, and arrayed into a tissue microarray using a Manual Tissue Arrayer (Servicebio, SH-30) with 3.0 mm cores; 3 cores per tumor were evenly distributed across the array. Serial 3 μm sections were cut onto charged slides for H&E staining (Mayer’s hematoxylin, Sigma-Aldrich; 0.1 % sodium bicarbonate; eosin Y solution, Sigma-Aldrich) and for immunostaining of Pkd1 (Abcam, ab74115; Proteintech, 22263-1-AP) and Ccl2 (Abcam, ab315478).

### Statistics.

The data distribution was characterized by frequency tabulation and summary statistics. Two-tailed Student’s *t* test, 1-way ANOVA, the Mann-Whitney Wilcoxon test, and the Kruskal-Wallis test were used to compare differences in the continuous variables and ordered categorical variables, whereas Pearson’s χ^2^ test and Fisher’s exact test were used for comparisons of the unordered categorical variables. For experiments with more than 2 groups, statistical significance was determined by 1-way ANOVA followed by Dunnett’s test. A *P* value less than 0.05 was considered statistically significant. All analyses were performed via the R package version 3.4.2 (https://cran.r-project.org/).

### Study approval.

All experiments were performed according to the Declaration of Helsinki. All tissue samples included in the present study were approved by the FUSCC Ethics Committee, and all patients provided written informed consent. All animal experiments were performed in compliance with the NIH’s *Guide for the Care and Use of Laboratory Animals* (National Academies Press, 2011) and were approved by the FUSCC Animal Ethics Committee (IACUC no., FUSCC-IACUC-2021479 and FUSCC-IACUC-2021478).

### Data availability.

The clinical datasets generated during and/or analyzed during the current study are available upon request from the corresponding author for research only, noncommercial purposes. The NGS data have been submitted to the National Omics Data Encyclopedia (NODE, https://www.biosino.org/node) under accessions OEZ00021764 and OEZ00021765, and the code is hosted on GitHub (https://github.com/yifanzhou330/mTNBC-PKD1/commit/165c21342c2d0b8e162231bca01c09352ae34426). Values for all data points in graphs are reported in the Supporting Data Values file.

## Author contributions

Conceptualization: XJ, YZJ, and ZHW. Methodology: XZZ, YFZ, and XJ. Data curation: YFZ, XHY, and XHD. Formal analysis: XZZ, YFZ, and XHY. Visualization: XZZ and KYZ. Writing of the original draft: XJ, XZZ, and YYW. Project administration: XZZ, YFZ, XJ, ZMS, YZJ, and ZHW. Supervision: XJ, YZJ, and ZHW.

## Funding support

- National Natural Science Foundation of China, grant/award 82103039, 82473339, and 82503865.
- Program of Shanghai Academic/Technology Research Leader, grant/award 20XD1421100.
- Shanghai Anti-Cancer Association, grant/award SACA-AX202314 and SACA-CY24A02.
- Foundation of the Shanghai Municipal Education Commission, grant/award 24RGZNA03.
- Natural Science Foundation of Shanghai, grant/award 25ZR1402088.

## Supplementary Material

Supplemental data

Unedited blot and gel images

Supplemental table 1

Supporting data values

## Version 1 03/02/2026

Electronic publication

## References

[1] Foulkes WD (2010). Triple-negative breast cancer. N Engl J Med.

[2] Bianchini G (2016). Triple-negative breast cancer: challenges and opportunities of a heterogeneous disease. Nat Rev Clin Oncol.

[3] Park S (2012). Characteristics and outcomes according to molecular subtypes of breast cancer as classified by a panel of four biomarkers using immunohistochemistry. Breast.

[4] Zhu X (2024). Stability and variability of molecular subtypes: comparative analysis of primary and metastatic triple-negative breast cancer. Cancer Biol Med.

[5] Seah DS (2014). Use and duration of chemotherapy in patients with metastatic breast cancer according to tumor subtype and line of therapy. J Natl Compr Canc Netw.

[6] Hu XC (2015). Cisplatin plus gemcitabine versus paclitaxel plus gemcitabine as first-line therapy for metastatic triple-negative breast cancer (CBCSG006): a randomised, open-label, multicentre, phase 3 trial. Lancet Oncol.

[7] Emens LA (2021). Atezolizumab and nab-Paclitaxel in advanced triple-negative breast cancer: biomarker evaluation of the IMpassion130 Study. J Natl Cancer Inst.

[8] Jiang YZ (2019). Genomic and transcriptomic landscape of triple-negative breast cancers: subtypes and treatment strategies. Cancer Cell.

[9] Lehmann BD (2011). Identification of human triple-negative breast cancer subtypes and preclinical models for selection of targeted therapies. J Clin Invest.

[10] Pusztai L (2024). Event-free survival by residual cancer burden with pembrolizumab in early-stage TNBC: exploratory analysis from KEYNOTE-522. Ann Oncol.

[11] Robson M (2017). Olaparib for metastatic breast cancer in patients with a germline BRCA mutation. N Engl J Med.

[12] Bardia A (2021). Sacituzumab govitecan in metastatic triple-negative breast cancer. N Engl J Med.

[13] (2022). Overall survival in the OlympiA phase III trial of adjuvant olaparib in patients with germline pathogenic variants in BRCA1/2 and high-risk, early breast cancer. Ann Oncol.

[14] Schmid P (2020). Capivasertib plus paclitaxel versus placebo plus paclitaxel as first-line therapy for metastatic triple-negative breast cancer: The PAKT Trial. J Clin Oncol.

[15] Desmedt C (2016). Genomic characterization of primary invasive lobular breast cancer. J Clin Oncol.

[16] Lindström LS (2012). Clinically used breast cancer markers such as estrogen receptor, progesterone receptor, and human epidermal growth factor receptor 2 are unstable throughout tumor progression. J Clin Oncol.

[17] Karlsson E (2014). Breast cancer during follow-up and progression - A population based cohort on new cancers and changed biology. Eur J Cancer.

[18] Ullah I (2018). Evolutionary history of metastatic breast cancer reveals minimal seeding from axillary lymph nodes. J Clin Invest.

[19] Bertucci F (2019). Genomic characterization of metastatic breast cancers. Nature.

[20] Naxerova K (2017). Origins of lymphatic and distant metastases in human colorectal cancer. Science.

[21] Brastianos PK (2015). Genomic characterization of brain metastases reveals branched evolution and potential therapeutic targets. Cancer Discov.

[22] Tanaka Y (2021). Multi-omic profiling of peritoneal metastases in gastric cancer identifies molecular subtypes and therapeutic vulnerabilities. Nat Cancer.

[23] Yaeger R (2018). Clinical sequencing defines the genomic landscape of metastatic colorectal cancer. Cancer Cell.

[24] Garcia-Recio S (2023). Multiomics in primary and metastatic breast tumors from the AURORA US network finds microenvironment and epigenetic drivers of metastasis. Nat Cancer.

[25] Garcia-Recio S (2020). FGFR4 regulates tumor subtype differentiation in luminal breast cancer and metastatic disease. J Clin Invest.

[26] Yates LR (2017). Genomic evolution of breast cancer metastasis and relapse. Cancer Cell.

[27] Holowatyj AN (2023). Racial/ethnic and sex differences in somatic cancer gene mutations among patients with early-onset colorectal cancer. Cancer Discov.

[28] Keenan T (2015). Comparison of the genomic landscape between primary breast cancer in african american versus white women and the association of racial differences with tumor recurrence. J Clin Oncol.

[29] Razavi P (2018). The genomic landscape of endocrine-resistant advanced breast cancers. Cancer Cell.

[30] Aftimos P (2021). Genomic and transcriptomic analyses of breast cancer primaries and matched metastases in AURORA, the breast international group (BIG) molecular screening initiative. Cancer Discov.

[31] Priestley P (2019). Pan-cancer whole-genome analyses of metastatic solid tumours. Nature.

[32] Lang GT (2020). Characterization of the genomic landscape and actionable mutations in Chinese breast cancers by clinical sequencing. Nat Commun.

[33] Nguyen B (2022). Genomic characterization of metastatic patterns from prospective clinical sequencing of 25,000 patients. Cell.

[34] Chaturantabut S (2019). Estrogen activation of g-protein-coupled estrogen receptor 1 regulates phosphoinositide 3-kinase and mTOR signaling to promote liver growth in zebrafish and proliferation of human hepatocytes. Gastroenterology.

[35] Garg P (2011). Notch1 regulates the effects of matrix metalloproteinase-9 on colitis-associated cancer in mice. Gastroenterology.

[36] Tang CK (2000). Epidermal growth factor receptor vIII enhances tumorigenicity in human breast cancer. Cancer Res.

[37] Gao Y (2025). NOTCH1 inhibition enhances immunogenicity and sensitizes triple-negative breast cancer to immune checkpoint inhibitors. Breast Cancer Res.

[38] Neamah AS Targeting mitochondrial metabolism to overcome hormone resistance in breast cancer. Naunyn Schmiedebergs Arch Pharmacol.

[39] Laughney AM (2020). Regenerative lineages and immune-mediated pruning in lung cancer metastasis. Nat Med.

[40] Subramanian A (2005). Gene set enrichment analysis: a knowledge-based approach for interpreting genome-wide expression profiles. Proc Natl Acad Sci U S A.

[41] Xiao Y (2019). Multi-omics profiling reveals distinct microenvironment characterization and suggests immune escape mechanisms of triple-negative breast cancer. Clin Cancer Res.

[42] Castanotto D (2009). The promises and pitfalls of RNA-interference-based therapeutics. Nature.

[43] Doudna JA (2014). Genome editing. The new frontier of genome engineering with CRISPR-Cas9. Science.

[44] Yang H (2020). CCL2-CCR2 axis recruits tumor associated macrophages to induce immune evasion through PD-1 signaling in esophageal carcinogenesis. Mol Cancer.

[45] Li X (2017). Targeting of tumour-infiltrating macrophages via CCL2/CCR2 signalling as a therapeutic strategy against hepatocellular carcinoma. Gut.

[46] McGranahan N (2015). Biological and therapeutic impact of intratumor heterogeneity in cancer evolution. Cancer Cell.

[47] Ginsburg O (2021). The role of genomics in global cancer prevention. Nat Rev Clin Oncol.

[48] Turajlic S (2016). Metastasis as an evolutionary process. Science.

[49] Bardia A (2019). Sacituzumab govitecan-hziy in refractory metastatic triple-negative breast cancer. N Engl J Med.

[50] Emens LA (2019). Long-term clinical outcomes and biomarker analyses of atezolizumab therapy for patients with metastatic triple-negative breast cancer: a phase 1 study. JAMA Oncol.

[51] Schmid P (2018). Atezolizumab and Nab-Paclitaxel in advanced triple-negative breast cancer. N Engl J Med.

[52] Shah SP (2012). The clonal and mutational evolution spectrum of primary triple-negative breast cancers. Nature.

[53] Balko JM (2014). Molecular profiling of the residual disease of triple-negative breast cancers after neoadjuvant chemotherapy identifies actionable therapeutic targets. Cancer Discov.

[54] Angus L (2019). The genomic landscape of metastatic breast cancer highlights changes in mutation and signature frequencies. Nat Genet.

[55] Ding L (2010). Genome remodelling in a basal-like breast cancer metastasis and xenograft. Nature.

[56] Shah SP (2009). Mutational evolution in a lobular breast tumour profiled at single nucleotide resolution. Nature.

[57] Ge LP (2024). ZNF689 deficiency promotes intratumor heterogeneity and immunotherapy resistance in triple-negative breast cancer. Cell Res.

[58] Pusztai L (2021). Durvalumab with olaparib and paclitaxel for high-risk HER2-negative stage II/III breast cancer: Results from the adaptively randomized I-SPY2 trial. Cancer Cell.

[59] Jia H (2017). Immunotherapy for triple-negative breast cancer: Existing challenges and exciting prospects. Drug Resist Updat.

[60] Schmid P (2024). Overall survival with pembrolizumab in early-stage triple-negative breast cancer. N Engl J Med.

[61] Schmid P (2022). Event-free survival with pembrolizumab in early triple-negative breast cancer. N Engl J Med.

[62] Schmid P (2020). Pembrolizumab for early triple-negative breast cancer. N Engl J Med.

[63] Torres VE (2019). Progress in the understanding of polycystic kidney disease. Nat Rev Nephrol.

[64] Bergmann C (2018). Polycystic kidney disease. Nat Rev Dis Primers.

[65] Sahin H (2010). Functional role of chemokines in liver disease models. Nat Rev Gastroenterol Hepatol.

[66] Li Y (2022). Recent advances in therapeutic strategies for triple-negative breast cancer. J Hematol Oncol.

[67] Jiang YZ (2021). Molecular subtyping and genomic profiling expand precision medicine in refractory metastatic triple-negative breast cancer: the FUTURE trial. Cell Res.

[68] Liu Y (2023). Subtyping-based platform guides precision medicine for heavily pretreated metastatic triple-negative breast cancer: The FUTURE phase II umbrella clinical trial. Cell Res.

[69] Chen L (2022). Famitinib with camrelizumab and nab-paclitaxel for advanced immunomodulatory triple-negative breast cancer (FUTURE-C-Plus): an open-label, single-arm, phase II Trial. Clin Cancer Res.

[70] Fan L (2024). Optimising first-line subtyping-based therapy in triple-negative breast cancer (FUTURE-SUPER): a multi-cohort, randomised, phase 2 trial. Lancet Oncol.

[71] Chavez A (2015). Highly efficient Cas9-mediated transcriptional programming. Nat Methods.

[72] Konermann S (2015). Genome-scale transcriptional activation by an engineered CRISPR-Cas9 complex. Nature.

[73] Zheng YZ (2018). PHF5A epigenetically inhibits apoptosis to promote breast cancer progression. Cancer Res.

[74] Zhu X (2021). Efficacy and mechanism of the combination of PARP and CDK4/6 inhibitors in the treatment of triple-negative breast cancer. J Exp Clin Cancer Res.

[75] Yang YL (2020). RNF144A functions as a tumor suppressor in breast cancer through ubiquitin ligase activity-dependent regulation of stability and oncogenic functions of HSPA2. Cell Death Differ.

[76] Ye FG (2015). Cytidine deaminase axis modulated by mir-484 differentially regulates cell proliferation and chemoresistance in breast cancer. Cancer Res.

[77] Chen L (2021). High Expression of microRNA-223 indicates a good prognosis in triple-negative breast cancer. Front Oncol.

