# Supplementary material for: Comprehensive genomic profiling of triple-negative breast cancer metastases identifies role of PKD1 in immunotherapy resistance
Source: J Clin Invest. 2026 Mar 2;136(5):e188989. doi: 10.1172/JCI188989 (PMC12948418; doi:10.1172/JCI188989)
Supplement: Supplemental data [file jci-136-188989-s207.pdf]

Figure S1

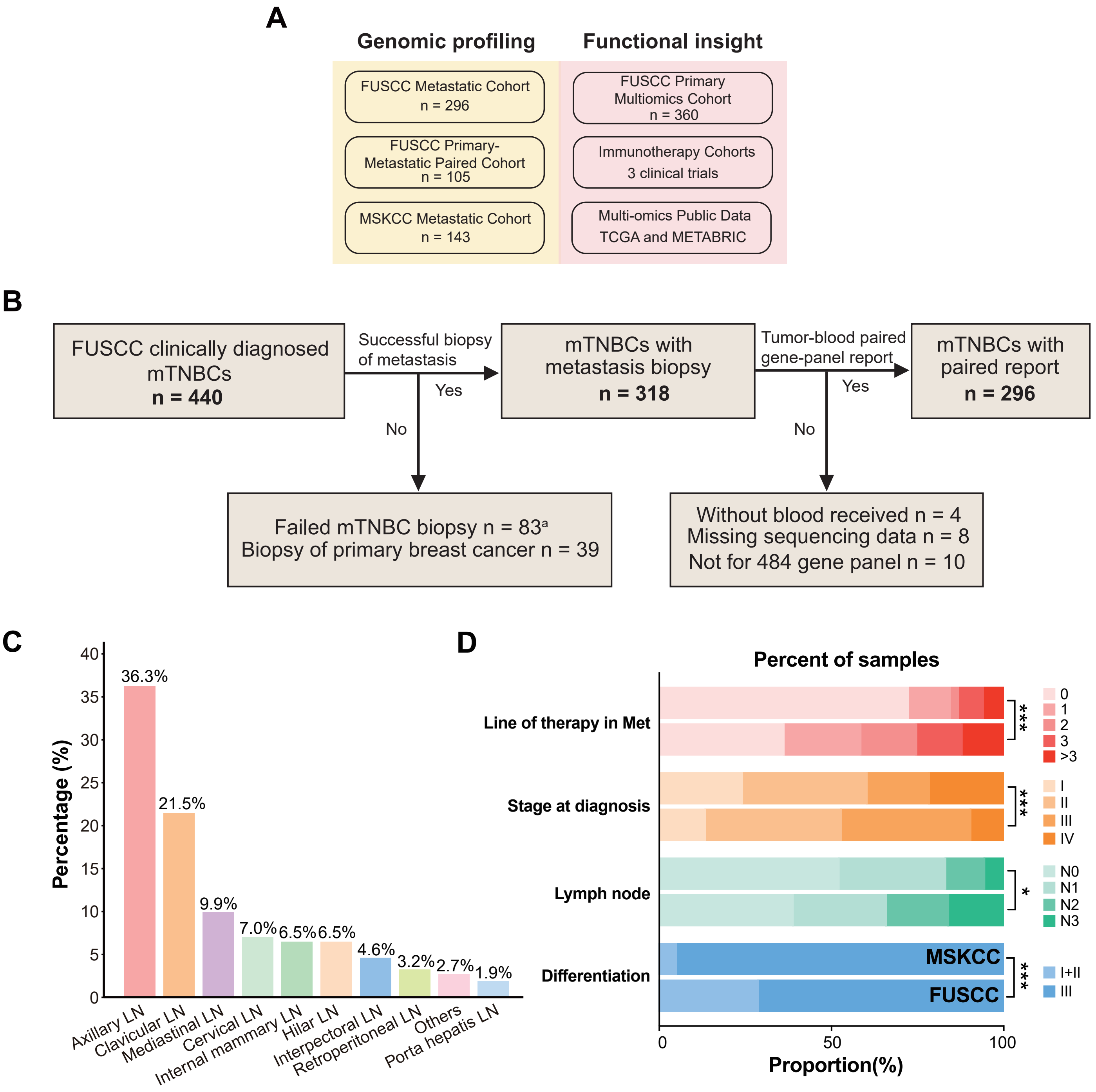

Figure S1 Study cohort description.

(A) Overview of the multi-cohorts used in our study.

(B) Flow chart of study population screening. [a], 44 patients did not undergo biopsy after registration; 14 biopsies showed no malignant evidence; 12 biopsies diagnosed second primary cancers; 8 biopsies confirmed non-TNBC breast cancer; 5 biopsies were classified as cancer of unknown primary.

(C) Distribution of 213 metastatic lymph node lesions related to Figure 1B. The lymph nodes analyzed here were not regional lymph nodes at initial diagnosis, but rather represented metastatic lymph node lesions that developed after radical resection.

(D) Clinical features of advanced TNBC in FUSCC and MSKCC. *P* values were calculated by the chi-square test.

Abbreviations: FUSCC, Fudan University Shanghai Cancer Center; TNBC, triple-negative breast cancer; mTNBC, metastatic TNBC; n, number; Met, metastasis; MSKCC, Memorial Sloan Kettering Cancer Center; TCGA, The Cancer Genome Atlas; METABRIC, Molecular Taxonomy of Breast Cancer International Consortium; LN, lymph node. Significant codes: ns, not significant; *, $p < 0.05$; **, $p < 0.01$; ***, $p < 0.001$.

Figure S2

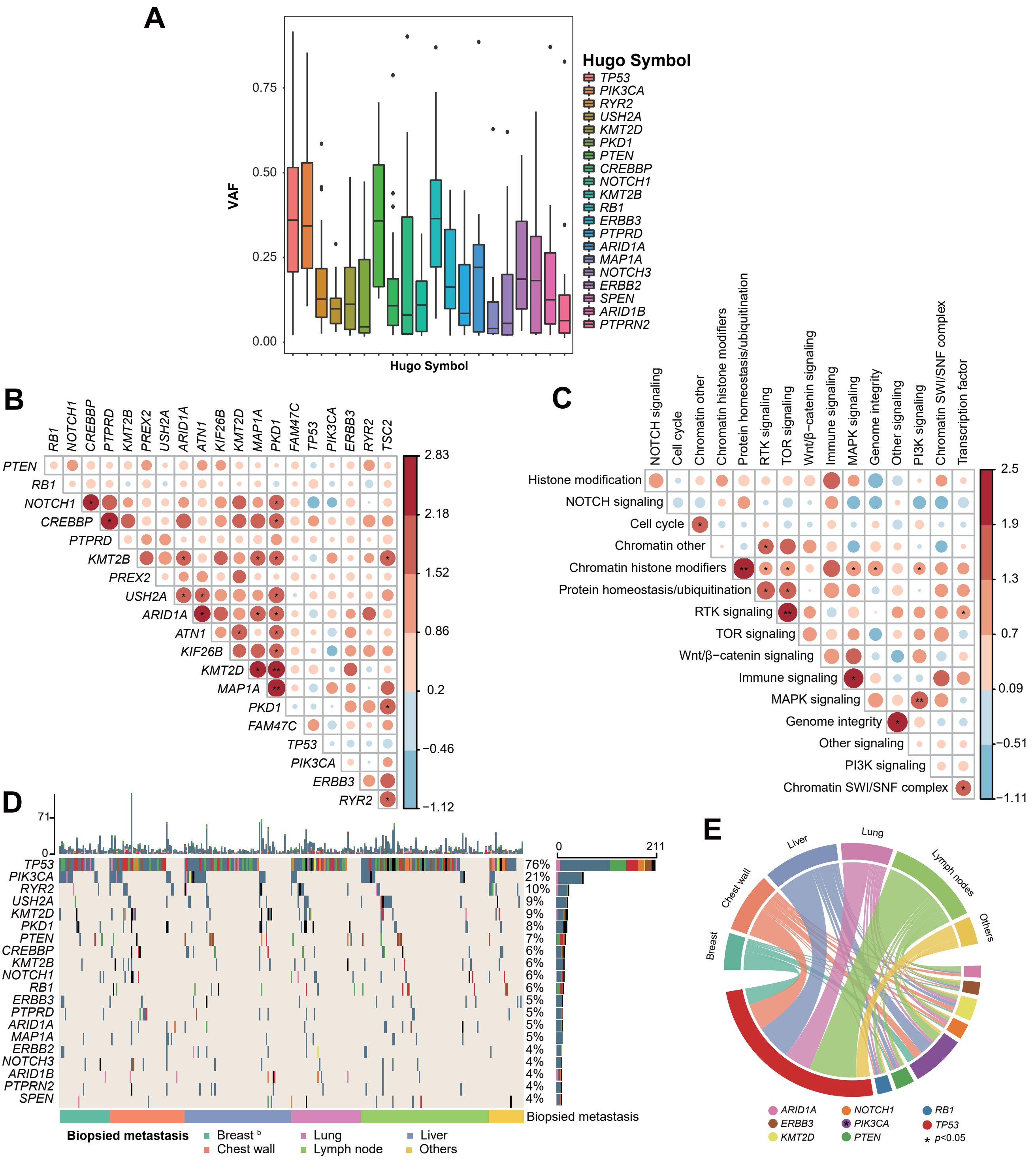

Figure S2 Genomic characteristics of triple negative breast cancer metastasis.

(A) The VAF of the top 20 genes in TNBC metastases in our cohort.

(B) Mutual exclusivity (blue) and co-occurrence (red) of gene mutations in the top 20 mutation frequency genes in our mTNBC cohort. Spectrum bar: log10 (odds ratio); the color intensity represents the scale of the value. *P* values were calculated using Fisher's exact test.

(C) Mutual exclusivity (blue) and co-occurrence (red) of mutation-associated pathways in our mTNBC cohort. Spectrum bar: log10 (odds ratio); the color intensity represents the scale of the value. *P* values were calculated using Fisher's exact test.

(D) The oncoplot showing the genomic features of specific organs grouped by metastases. [b],The "breast" subgroup in our metastatic analysis refers exclusively to metastatic lesions located in the breast tissue and are not primary breast cancer.

(E) Distribution of recurrently mutated genes in different metastases. Line thickness corresponds to the frequency of gene mutations arising in the indicated metastases. *P* values were calculated via Fisher's exact test.

Abbreviations: VAF, variant allele frequency; TNBC, triple-negative breast cancer. Significant codes: ns, not significant; *, $p < 0.05$; **, $p < 0.01$; ***, $p < 0.001$.

Figure S3

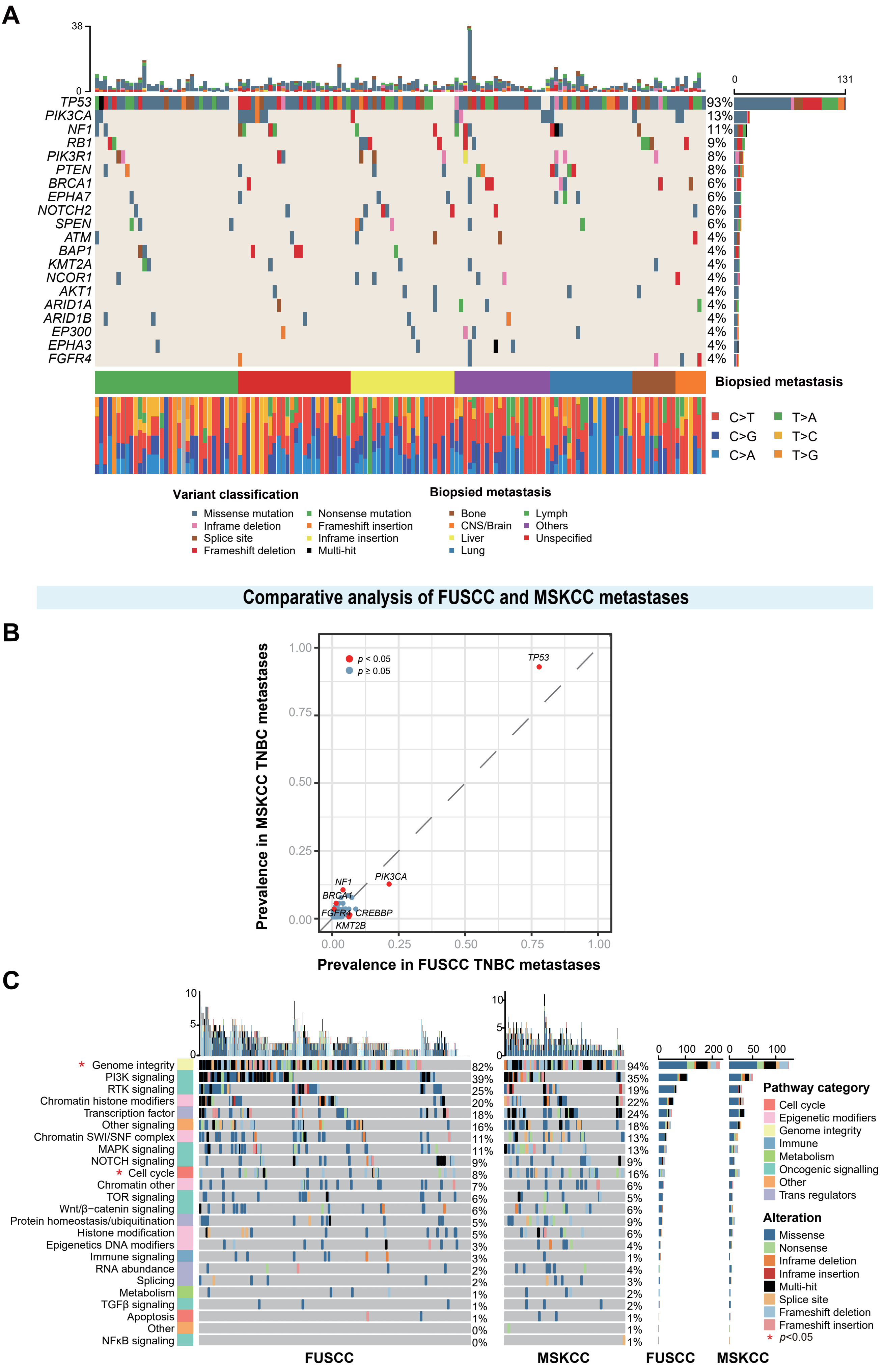
A
TP53
PIK3CA
NF1
RB1
PIK3R1
PTEN
BRCA1
EPHA7
NOTCH2
SPEN
ATM
BAP1
KMT2A
NCOR1
AKT1
ARID1A
ARID1B
EP300
EPHA3
FGFR4
93%
13%
11%
9%
8%
8%
6%
6%
6%
6%
4%
4%
4%
4%
4%
4%
4%
4%
4%
4%
38
0
0
131
Biopsied metastasis
C>T
C>G
C>A
T>A
T>C
T>G
Variant classification
Missense mutation
Inframe deletion
Splice site
Frameshift deletion
Nonsense mutation
Frameshift insertion
Inframe insertion
Multi-hit
Biopsied metastasis
Bone
CNS/Brain
Liver
Lung
Lymph
Others
Unspecified
Comparative analysis of FUSCC and MSKCC metastases
B
p < 0.05
p ≥ 0.05
TP53
PIK3CA
NF1
BRCA1
FGFR4
CREBBP
KMT2B
Prevalence in MSKCC TNBC metastases
Prevalence in FUSCC TNBC metastases
0.00
0.25
0.50
0.75
1.00
C
* Genome integrity
PI3K signaling
RTK signaling
Chromatin histone modifiers
Transcription factor
Other signaling
Chromatin SWI/SNF complex
MAPK signaling
NOTCH signaling
* Cell cycle
Chromatin other
TOR signaling
Wnt/β−catenin signaling
Protein homeostasis/ubiquitination
Histone modification
Epigenetics DNA modifiers
Immune signaling
RNA abundance
Splicing
Metabolism
TGFβ signaling
Apoptosis
Other
NFκB signaling
82%
39%
25%
20%
18%
16%
11%
11%
9%
8%
7%
6%
6%
5%
5%
3%
3%
2%
2%
1%
1%
1%
0%
0%
94%
35%
19%
22%
24%
18%
13%
13%
9%
16%
6%
5%
6%
9%
6%
4%
1%
4%
3%
2%
2%
1%
1%
1%
10
5
0
0
100
200
50
100
Pathway category
Cell cycle
Epigenetic modifiers
Genome integrity
Immune
Metabolism
Oncogenic signalling
Other
Trans regulators
Alteration
Missense
Nonsense
Inframe deletion
Inframe insertion
Multi-hit
Splice site
Frameshift deletion
Frameshift insertion
* p<0.05
FUSCC
MSKCC
FUSCC
MSKCC

Figure S3 Comparative genomic analysis of TNBC metastases between the FUSCC and MSKCC cohorts.

(A) Sequencing data of MSKCC advanced TNBC samples classified by mutation profile and annotated with the variation type and mutation frequency. The mutation counts in each sample and each gene are provided above and on the right side, respectively.

(B) Identification of population-specific genomic mutations in TNBC metastases by comparing the mutation frequencies in the FUSCC cohort and MSKCC cohort. *P* values were calculated via the chi-square test.

(C) Comparison of genomic mutation-associated signaling pathways between the FUSCC and MSKCC cohorts. *P* values were calculated via the chi-square test.

Abbreviations: MSKCC, Memorial Sloan Kettering Cancer Center; TNBC, triple-negative breast cancer; FUSCC, Fudan University Shanghai Cancer Center. Significant codes: ns, not significant; *, $p < 0.05$; **, $p < 0.01$; ***, $p < 0.001$.

Figure S4

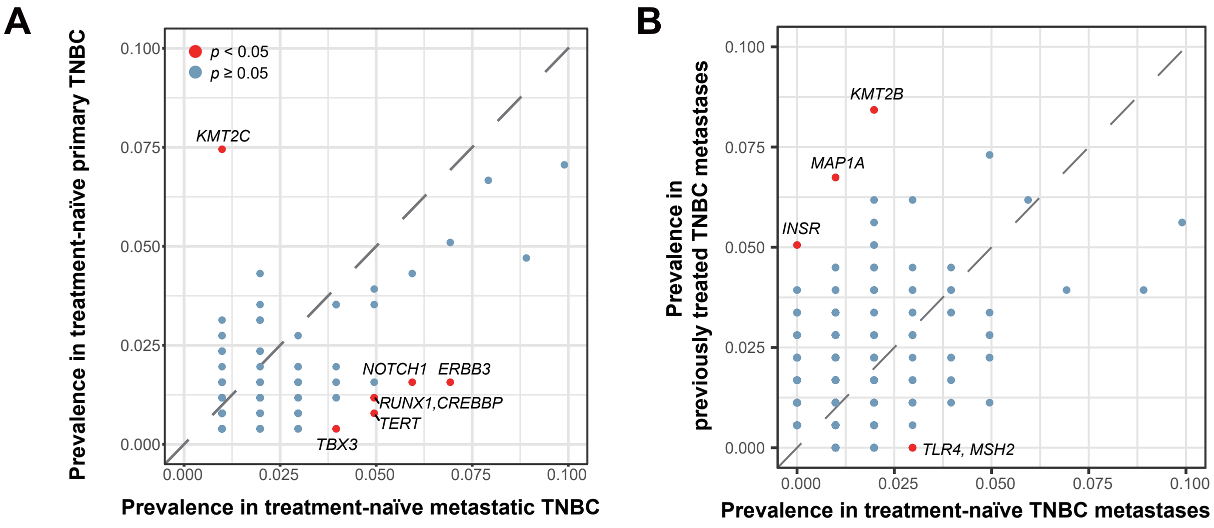

Figure S4 Comparative genomic analysis of primary and metastatic TNBC tumors in the FUSCC cohort.

(A) Comparison of genomic mutations between treatment-naïve primary tumors (n = 252) and metastases (n =101) in TNBC. *P* values were calculated by the chi-square test.

(B) Comparison of genomic mutations between treatment-naïve and previously treated TNBC metastases (n = 178). *P* values were calculated by the chi-square test.

Abbreviations: FUSCC, Fudan University Shanghai Cancer Center; TNBC, triple-negative breast cancer.

Figure S5

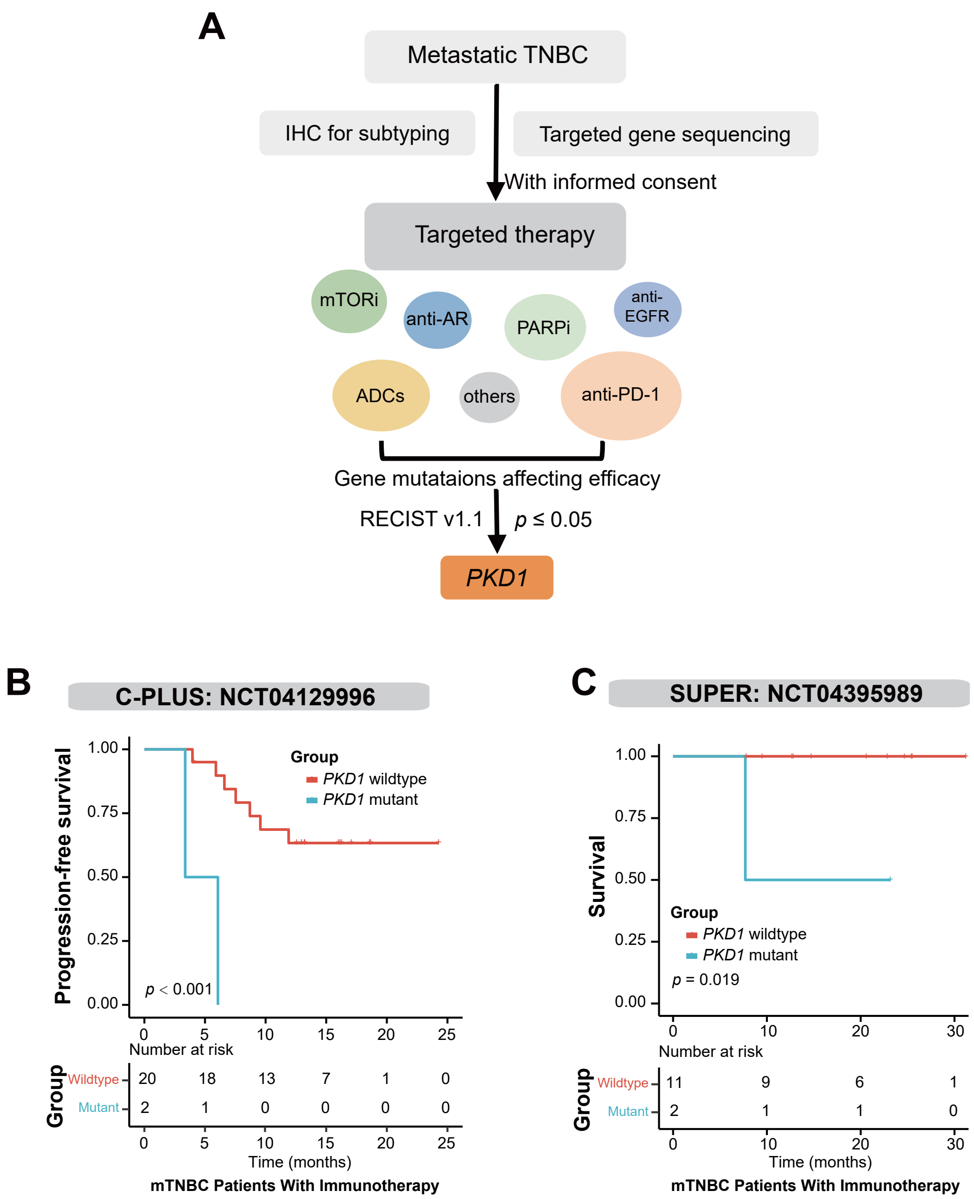

Figure S5 Genomic profiling of metastases informs TNBC precision therapy and identifies PKD1 as a potential biomarker for immunotherapy.

(A) Schematic overview of the genomic profiling of metastases informing TNBC precision therapy. Advanced TNBC patients underwent next-generation sequencing and received personalized treatment. Efficacy analysis identified potential biomarkers for treatment response.

(B-C) Survival of advanced TNBC patients receiving immunotherapy in the C-PLUS trial (B, NCT04129996) and the FUTURE-SUPER trial (C, NCT04395989). *P* values were calculated via the log-rank test.

Abbreviations: TNBC, triple-negative breast cancer; IHC, immunohistochemistry; AR, androgen receptor; PARPi, poly (ADP-ribose) polymerase inhibitor; EGFR, epidermal growth factor receptor; RECIST v1.1, Response Evaluation Criteria in Solid Tumors version 1.1.

Figure S6

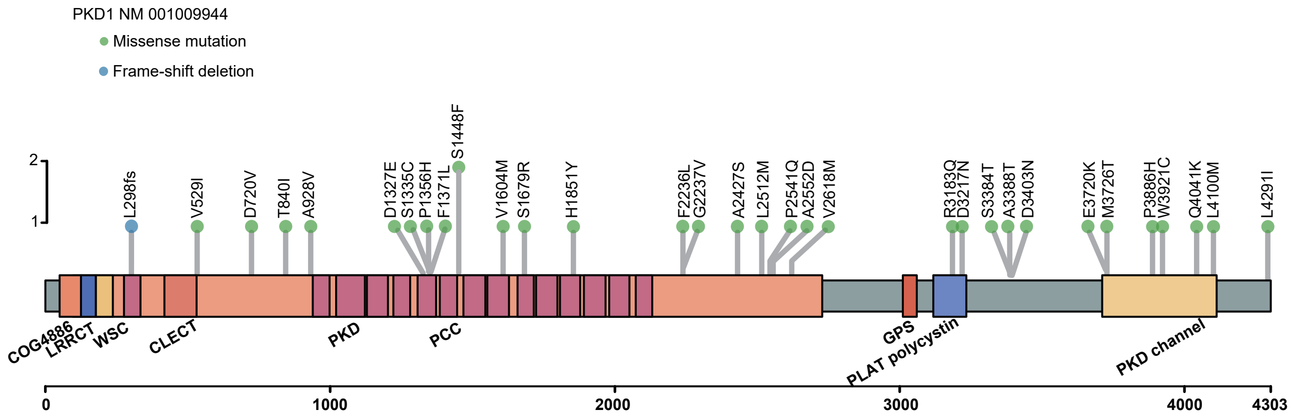

Figure S6 Somatic *PKD1* mutation sites of triple-negative breast cancer metastases in our cohort.

Figure S7

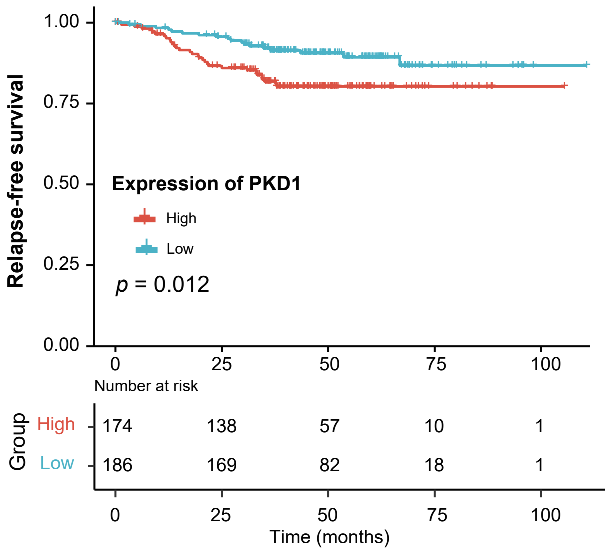

Figure S7 Relapse-free survival analysis of triple-negative breast cancer patients stratified by PKD1 expression levels in our clinical cohort. *P* values were calculated via the log-rank test.

Figure S8

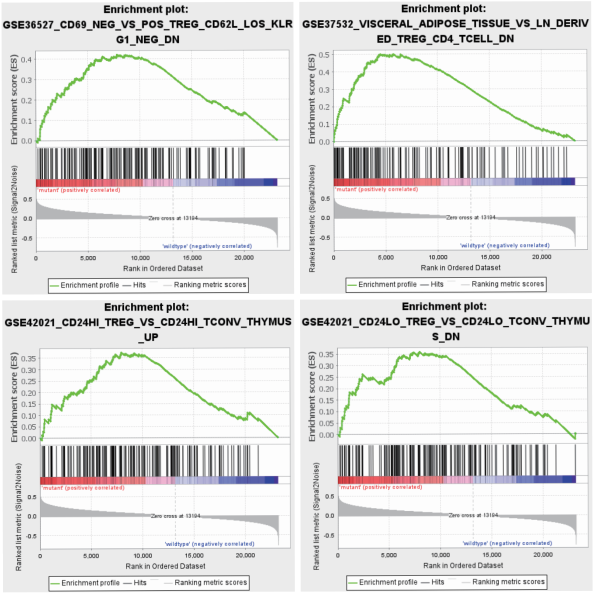

Figure S8 GSEA analysis showing that TNBCs with PKD1 mutations were enriched with numerous immune-negatively associated Treg signaling pathways.
Abbreviations: GSEA, gene set enrichment analysis; TNBCs, triple-negative breast cancers.

Figure S9

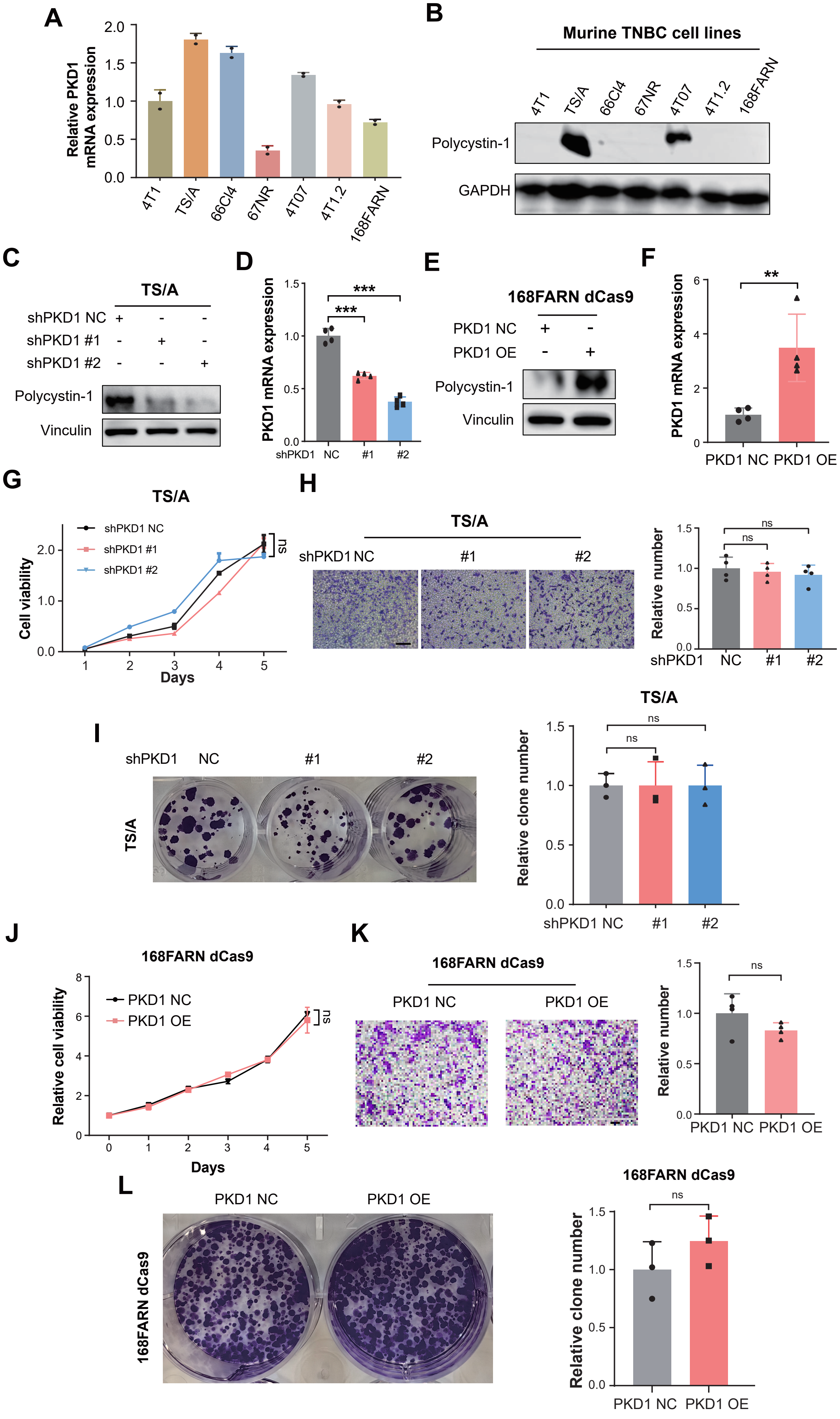
A
Relative PKD1 mRNA expression
4T1
TS/A
66Cl4
67NR
4T07
4T1.2
168FARN
B
Murine TNBC cell lines
Polycystin-1
GAPDH
C
TS/A
shPKD1 NC
shPKD1 #1
shPKD1 #2
Vinculin
D
PKD1 mRNA expression
***
E
168FARN dCas9
PKD1 NC
PKD1 OE
F
**
G
Cell viability
Days
H
Relative number
ns
I
Relative clone number
J
Relative cell viability
K
L

Figure S9 Effects of PKD1 on tumor proliferation and migration in vitro.
(A-B) PKD1 expression was detected by qRT-PCR (**A**) and WB (**B**) in seven mouse TNBC cell lines.
(C-D) Knockdown efficiency of PKD1 (encoding polycystin-1 protein) in TS/A cell line tested by WB (**C**) and RT-qPCR (**D**). *P* values were calculated by the Wilcoxon test.
(E-F) Overexpression efficiency of PKD1 (encoding polycystin-1 protein) in 168FARN cell line tested by WB (**E**) and RT-qPCR (**F**). *P* values were calculated by the Wilcoxon test.
(G) Growth curves of TS/A cells in the PKD1-KD and control groups. *P* values were calculated by the Wilcoxon test.
(H) Transwell migration assay of TS/A cells in the PKD1-KD and control groups. The migration time was 14 hours. The left panel shows representative images of transwell migration and the right panel displays quantification of transwell migration for the control and PKD1-KD groups. *P* values were calculated by the Wilcoxon test. Scale bar: 200 μm.
(I) Clone formation of TS/A cells in the PKD1-KD and control groups. The left panel shows representative images of clone formation and the right panel displays quantification of clone formation for the control and PKD1-KD groups. *P* values were calculated by the Wilcoxon test.
(J) Growth curves of 168FARN cells in the PKD1-OE and control groups. *P* values were calculated by the Wilcoxon test.
(K) Transwell migration assay of 168FARN cells in the PKD1-OE and control groups. The migration time was 24 hours. The left panel shows representative images of transwell migration and the right panel displays quantification of transwell migration for control and PKD1-OE groups. *P* values were calculated by the Wilcoxon test.Scale bar: 500 μm.
(L) Clone formation of 168FARN cells in the PKD1-OE and control groups. The left panel shows representative images of clone formation and the right panel displays quantification of clone formation for the control and PKD1-OE groups. *P* values were calculated by the Wilcoxon test.
Abbreviations: WB, western blotting; RT-qPCR, quantitative real time polymerase chain reaction; KD, knockdown; OE, overexpression; Significant codes: ns, not significant; *, $p < 0.05$; **, $p < 0.01$; ***, $p < 0.001$.

Figure S10

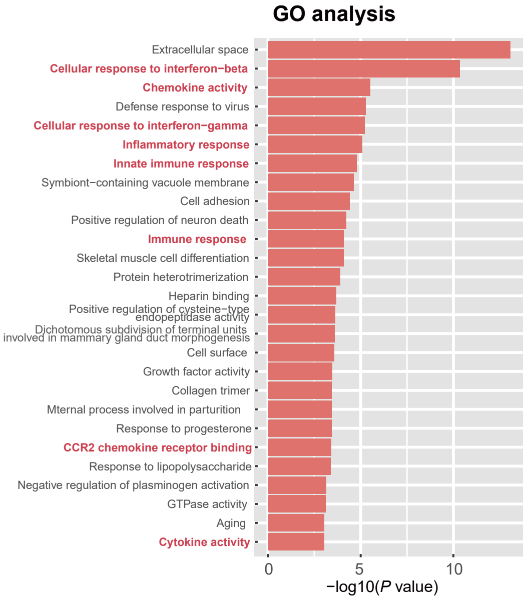

Figure S10 GO analysis showing that low expression of PKD1 was significantly enriched in multiple macrophage-related pathways.
