# Supplementary material for: Comprehensive genomic profiling of triple-negative breast cancer metastases identifies role of PKD1 in immunotherapy resistance
Source: J Clin Invest. 2026 Mar 2;136(5):e188989. doi: 10.1172/JCI188989 (PMC12948418; doi:10.1172/JCI188989)
Supplement: Unedited blot and gel images [file jci-136-188989-s208.pdf]

Figure 5A

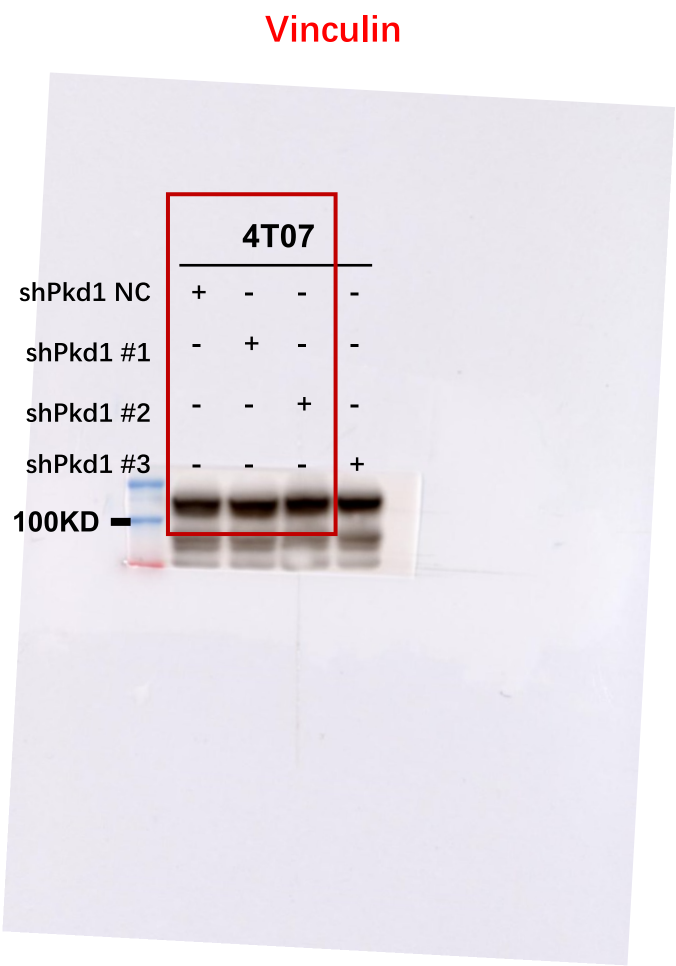

Vinculin
4T07
shPkd1 NC + - - -
shPkd1 #1 - + - -
shPkd1 #2 - - + -
shPkd1 #3 - - - +
100KD

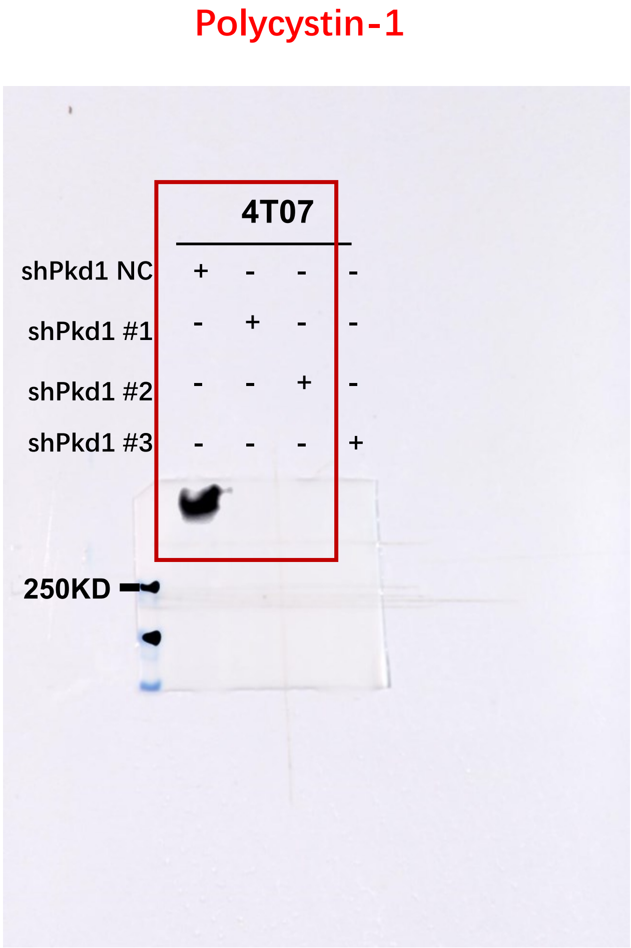

Polycystin-1
4T07
shPkd1 NC + - - -
shPkd1 #1 - + - -
shPkd1 #2 - - + -
shPkd1 #3 - - - +
250KD

Figure 5F

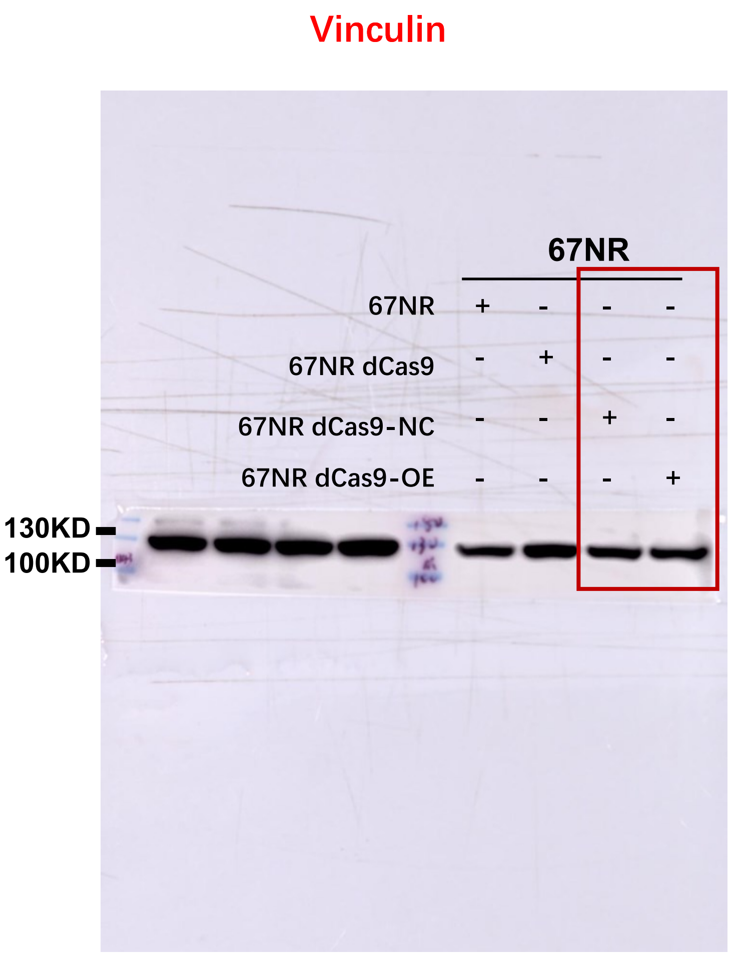
Vinculin
67NR
67NR + - - -
67NR dCas9 - + - -
67NR dCas9-NC - - + -
67NR dCas9-OE - - - +
130KD
100KD

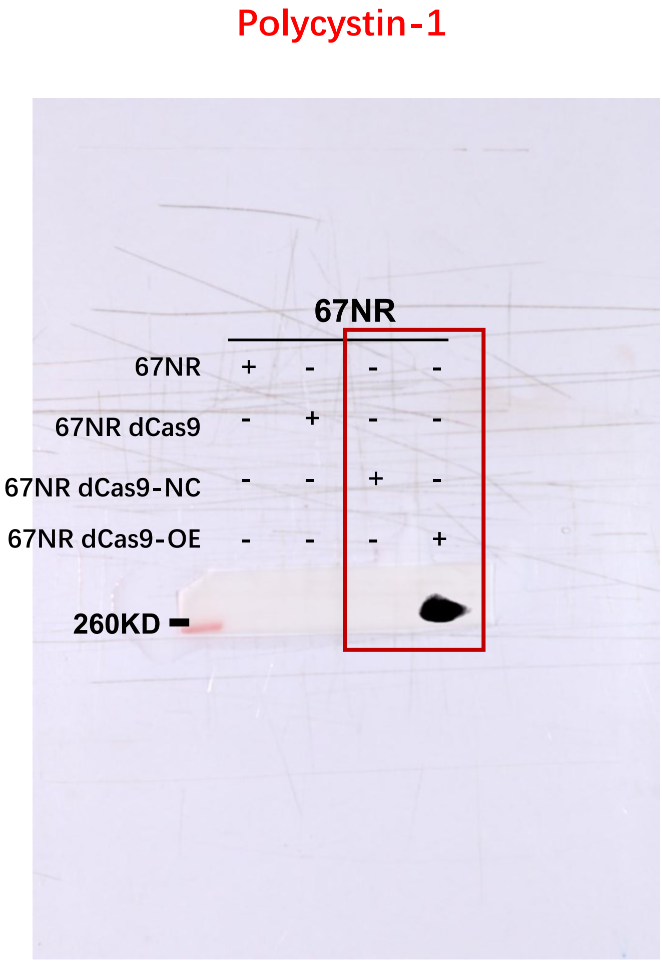
Polycystin-1
67NR
67NR + - - -
67NR dCas9 - + - -
67NR dCas9-NC - - + -
67NR dCas9-OE - - - +
260KD

Figure S9B

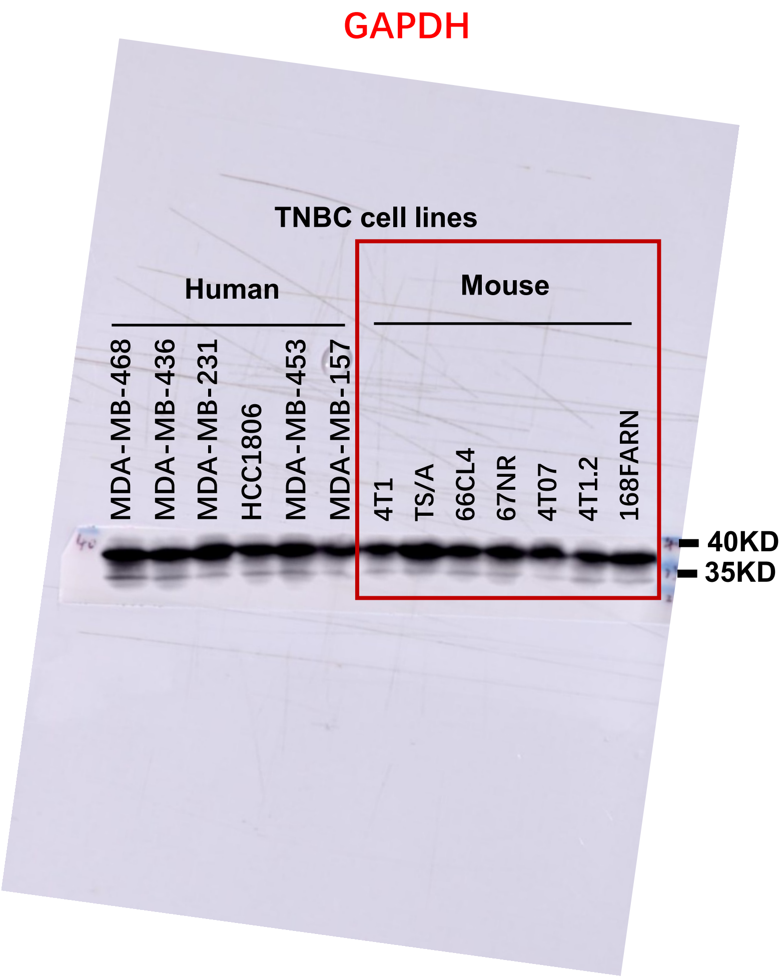

GAPDH
TNBC cell lines
Human
Mouse
MDA-MB-468
MDA-MB-436
MDA-MB-231
HCC1806
MDA-MB-453
MDA-MB-157
4T1
TS/A
66CL4
67NR
4T07
4T1.2
168FARN
40KD
35KD

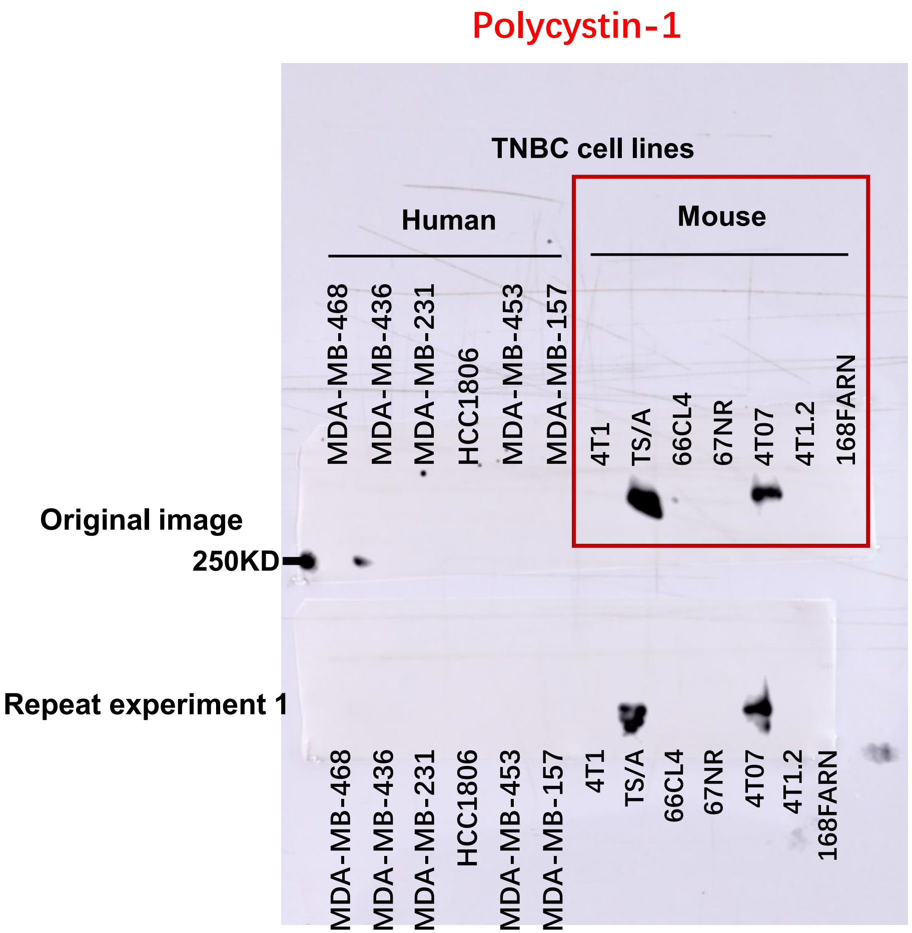

Polycystin-1
TNBC cell lines
Human
Mouse
MDA-MB-468
MDA-MB-436
MDA-MB-231
HCC1806
MDA-MB-453
MDA-MB-157
4T1
TS/A
66CL4
67NR
4T07
4T1.2
168FARN
Original image
250KD
Repeat experiment 1
MDA-MB-468
MDA-MB-436
MDA-MB-231
HCC1806
MDA-MB-453
MDA-MB-157
4T1
TS/A
66CL4
67NR
4T07
4T1.2
168FARN

Figure S9C

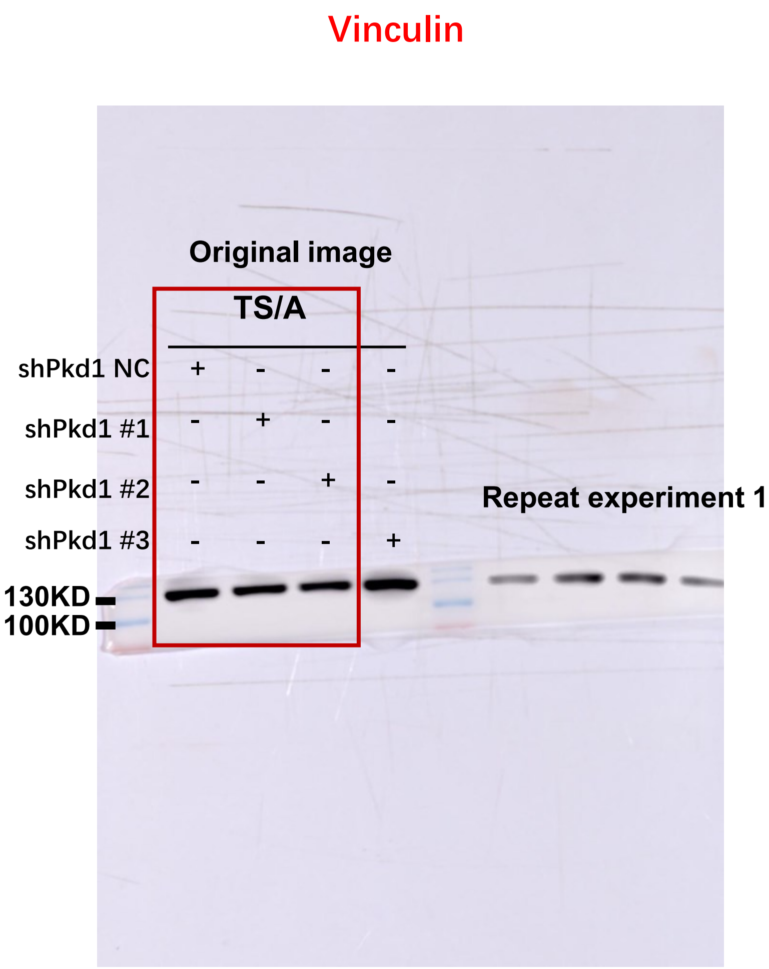

Vinculin
Original image
TS/A
shPkd1 NC + - - -
shPkd1 #1 - + - -
shPkd1 #2 - - + -
shPkd1 #3 - - - +
Repeat experiment 1
130KD
100KD

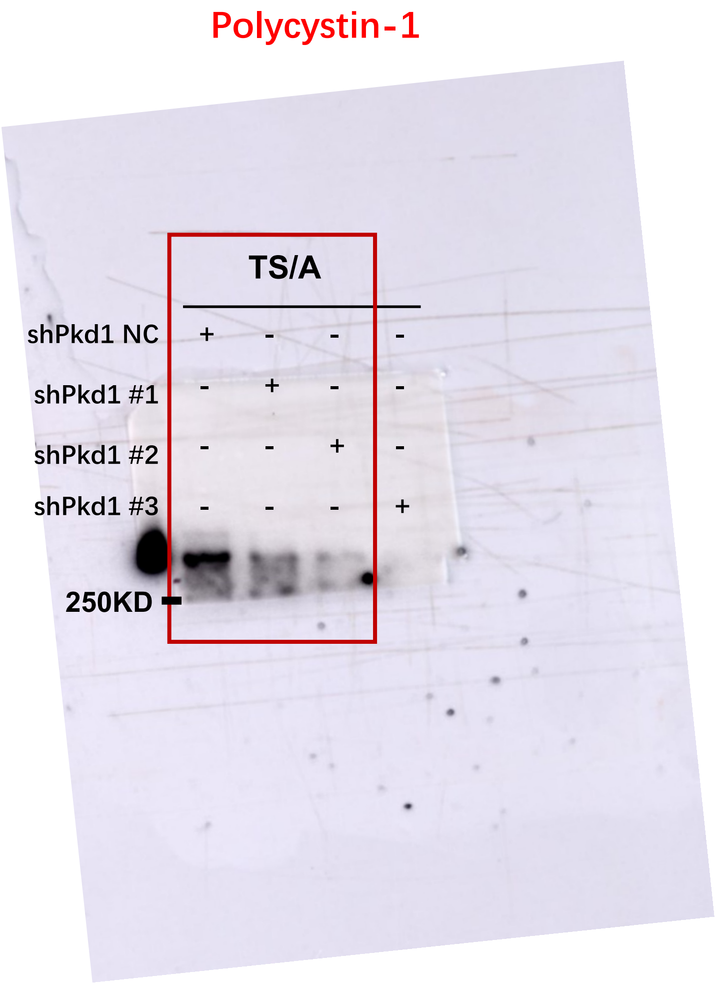

Polycystin-1
TS/A
shPkd1 NC + - - -
shPkd1 #1 - + - -
shPkd1 #2 - - + -
shPkd1 #3 - - - +
250KD

Figure S9E

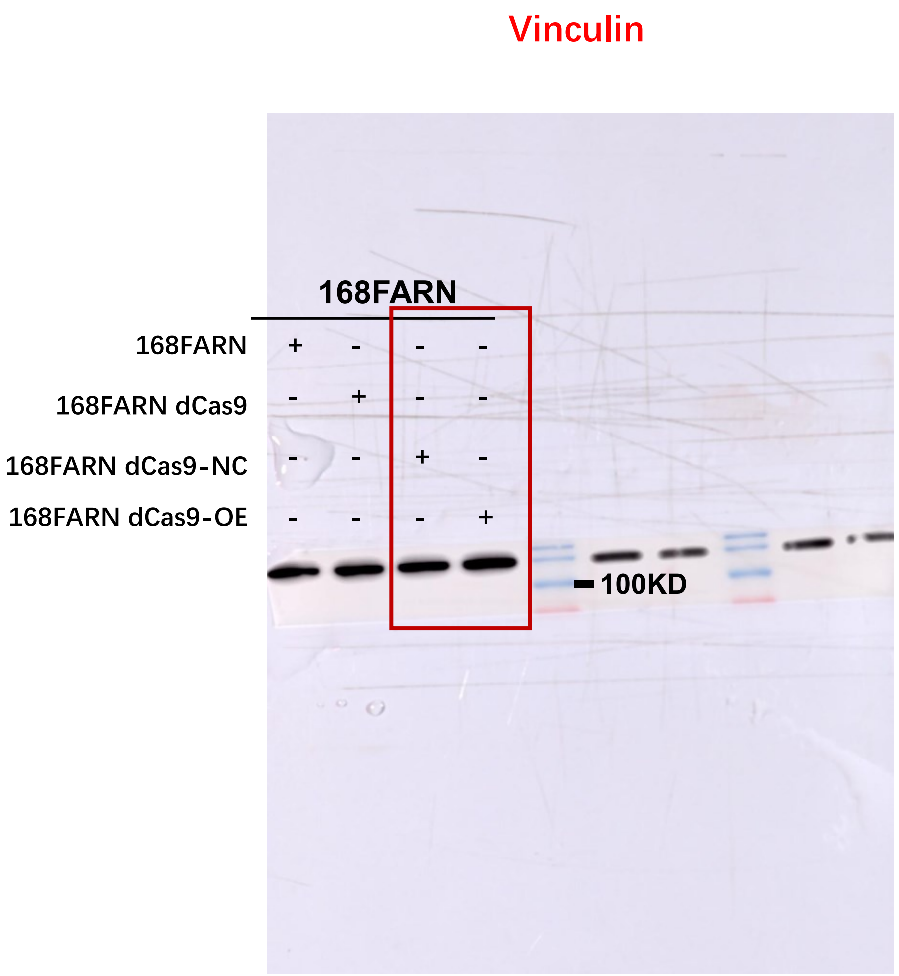
Vinculin
168FARN
168FARN + - - -
168FARN dCas9 - + - -
168FARN dCas9-NC - - + -
168FARN dCas9-OE - - - +
100KD

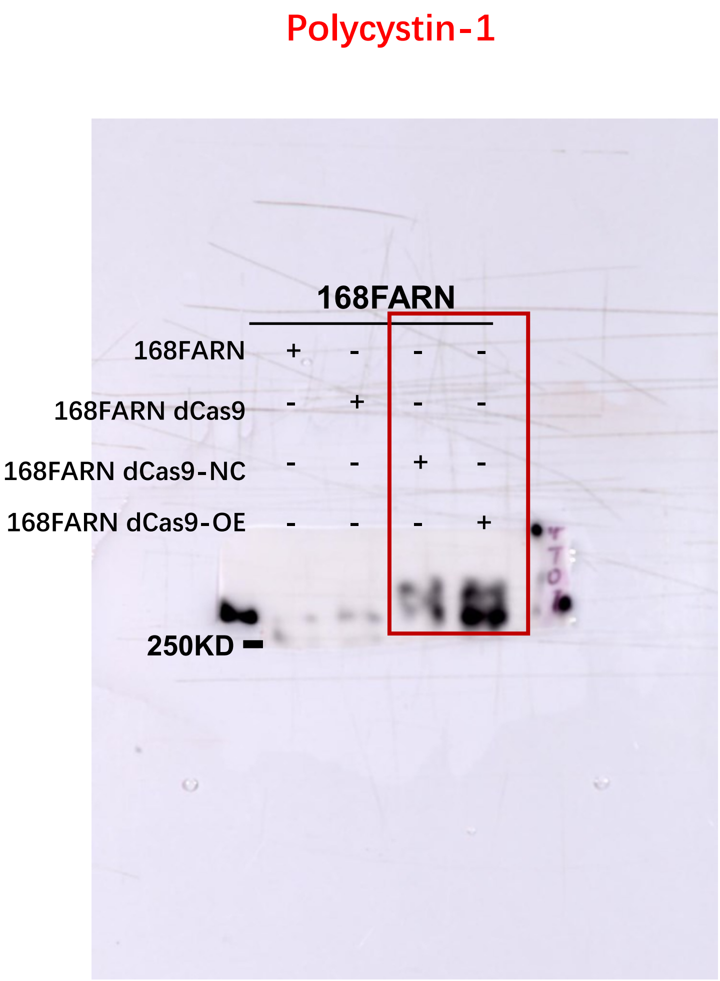
Polycystin-1
168FARN
168FARN + - - -
168FARN dCas9 - + - -
168FARN dCas9-NC - - + -
168FARN dCas9-OE - - - +
250KD
